# Therapeutic Potential of Plants and Plant Derived Phytochemicals against Acetaminophen-Induced Liver Injury

**DOI:** 10.3390/ijms19123776

**Published:** 2018-11-28

**Authors:** Sandeep B. Subramanya, Balaji Venkataraman, Mohamed Fizur Nagoor Meeran, Sameer N. Goyal, Chandragouda R. Patil, Shreesh Ojha

**Affiliations:** 1 Department of Physiology, College of Medicine and Health Sciences, PO Box # 17666, United Arab Emirates University, Al Ain 17666, UAE; sandeep.bs@uaeu.ac.ae (S.B.S.); balajiv@uaeu.ac.ae (B.V.); 2 Department of Pharmacology and Therapeutics, College of Medicine and Health Sciences, PO Box # 17666, United Arab Emirates University, Al Ain 17666, UAE; nagoormeeran1985@uaeu.ac.ae; 3 Department of Pharmacology, SVKM’s Institute of Pharmacy, Dhule, Maharashtra 424 001, India; goyal.aiims@gmail.com; 4 Department of Pharmacology, R. C. Patel Institute of Pharmaceutical Education and Research, Shirpur, Dhule, Maharashtra 425 405, India; pchandragouda@yahoo.com

**Keywords:** APAP, acetaminophen, hepatotoxicity, hpatoprotection, paracetamol, animals, preclinical studies, natural products, small molecules, phytochemicals, plants

## Abstract

Acetaminophen (APAP), which is also known as paracetamol or *N*-acetyl-*p*-aminophenol is a safe and potent drug for fever, pain and inflammation when used at its normal therapeutic doses. It is available as over-the-counter drug and used by all the age groups. The overdose results in acute liver failure that often requires liver transplantation. Current clinical therapy for APAP-induced liver toxicity is the administration of *N*-acetyl-cysteine (NAC), a sulphydryl compound an approved drug which acts by replenishing cellular glutathione (GSH) stores in the liver. Over the past five decades, several studies indicate that the safety and efficacy of herbal extracts or plant derived compounds that are used either as monotherapy or as an adjunct therapy along with conventional medicines for hepatotoxicity have shown favorable responses. Phytochemicals mitigate necrotic cell death and protect against APAP-induced liver toxicityby restoring cellular antioxidant defense system, limiting oxidative stress and subsequently protecting mitochondrial dysfunction and inflammation. Recent experimental evidences indicat that these phytochemicals also regulate differential gene expression to modulate various cellular pathways that are implicated in cellular protection. Therefore, in this review, we highlight the role of the phytochemicals, which are shown to be efficacious in clinically relevant APAP-induced hepatotoxicity experimental models. In this review, we have made comprehensive attempt to delineate the molecular mechanism and the cellular targets that are modulated by the phytochemicals to mediate the cytoprotective effect against APAP-induced hepatotoxicity. In this review, we have also defined the challenges and scope of phytochemicals to be developed as drugs to target APAP-induced hepatotoxicity.

## 1. Introduction

Acetaminophen (APAP), which is also known as paracetamol or *N*-acetyl-*p*-aminophenol appears as a safe and potent drug for fever, pain and inflammation at its normal therapeutic doses. It is indicated for all age groups and is often available as over-the-counter medicine. However, the overdoses of APAP whether intentional or unintentional may cause dose-dependent acute liver failure, a potentially fatal liver necrosis that has limited treatment options except liver transplantation [1]. APAP is metabolized by sulfation and glucuronidation in liver with less than 5–10% being metabolized by the hepatic cytochrome P450 (CYP450) system. Simultaneously, glutathione redox system also plays an important role for inactivating the formed metabolite by glutathione conjugation that leads to the consumption of GSH in the liver. The rapid conversion of APAP in to the reactive metabolite *N*-acetyl-*p*-benzoquinone imine (NAPQI) by CYP450 enzymes, mainly CYP2E1 results in the generation of free radicals and which binds covalently with the cellular nucleophiles such as DNA, RNA and proteins that leads cell death [2,3]. Although, NAPQI is detoxified by GSH which results in depletion of the cellular GSH stores and formation of protein adducts. Oxidative stress in the mitochondria further leads to the activation of the enzymes of signaling cascade such as redox-sensitive MAP kinases and the phosphorylation of c-jun-*N*-terminal kinase (JNK) [4,5,6,7,8,9]. This mitochondrial oxidative and nitrosative stress initiates the overt activation of mitochondrial permeability transition (MPT) and it causes interruption and destabilizes the membrane potential which leads mitochondrial swelling and rupture of the membranes [10,11]. Following the rupture of the membrane, there is a massive release of apoptosis-inducing factor (AIF), endonuclease G and caspases activators into the cytoplasm and concomitant tranlocation into the nucleus to initiate nuclear DNA fragmentation [5,12]. Together, mitochondrial impairment along with DNA fragmentation are the prime cause of hepatocyte necrosis observed in APAP-induced liver toxicity [13].

Biochemically, CYP450, the heme-containing monoxygenases that are predominantly present in liver is believed to play a regulatory and catalytic activity in the metbolism of APAP and represent an important therapeutic target for metabolic modulation [14]. Among the various isoforms of CYP450 (CYP1A1, CYP1A2, CYP1B1, CYP2A6, CYP2E1 and CYP3A), CYP2E1 is the major mediator of APAP-induced bioactivation [15]. It appears critical to NAPQI formation and have shown to contribute to 30–78% of APAP metabolism [16]. Furthermore, CYP2E1 mediates NADPH oxidase activity resulting in the generation of free radicals that leads to hepatic injury. Therefore, inhibiting CYP2E1 activity can affect APAP metabolism and represent a plausible pharmacological mechanism for therapeutic interventions for APAP-induced liver injury [17,18,19].

**Figure 1:**
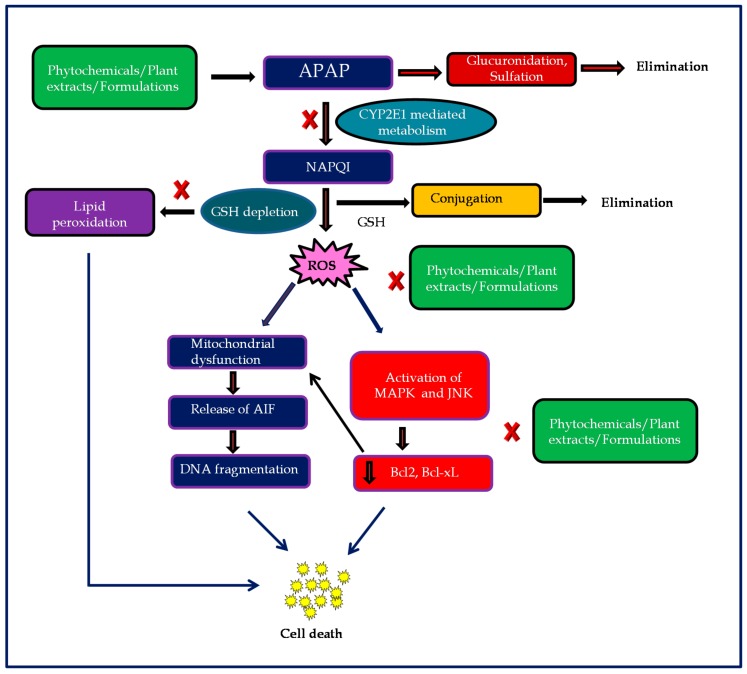
Schematic representation of phytochemical attenuate acetaminophen-induced liver toxicity.

Excessive metabolites formation depletes GSH from liver, therefore the treatment of APAP toxicity is based on replenishing GSH stores in the liver by the use of glutathione precursor; *N*-acetylcysteine (NAC), a sulphydryl compound [20]. NAC is the only drug approved and available as an antidote for APAP-induced hepatotoxicity, that seems to be effective only when it is administered either orally or intravenously within 10h of APAP overdose [21]. Therefore, it is reasonable to conceive that the inhibitors of glutathione synthesis will exacerbate APAP-induced hepatotoxicity and the precursor of GSH formation will be hepatoprotective against APAP. The graphical representation relevant to the hepatic injury that is caused by APAP metabolism as well as the efficaciousness of the phytochemicals or plant extracts/formulations is depicted in Figure 1.

Over the past five decades, the safety and efficacy of several herbal extracts and plant derived compounds either as monotherapy or as an adjunct to conventional medicines for APAP-induced hepatotoxicity appears to be favorable due to their ability to limit APAP-induced hepatotoxicity. Since the recognition of APAP associated liver toxicity in 1960s, experimental models including both in vivo (animal models) and in vitro (cell lines) have been employed for screening hepatoprotective properties [22,23] either of synthetic origin or plant derived natural extracts. Many experimental studies carried out using natural products have shown heptoprotective properties in clinically relevant APAP-induced liver toxicity model [24]. Therefore, this review renders an account of all the plant derived natural compounds, namely, phytochemicals that have been shown efficacious in APAP-induced hepatotoxicity.

This review summarized the current literature and comprehensively discussed the hepatoprotective properties of all the phytochemicals that were investigated in APAP-induced hepatotoxicity. This review also assessed the challenges and scope of the phytochemicals that can be developed as potential new hepatoprotective drugs for APAP-induced hepatotoxicity. The details of each phytochemical showed hepatoprotective are represented in the individual paragraphs. The dose of the phytochemicals investigated, the regimen of APAP used to induce liver toxicity in the in vivo or in vitro models and the parameters assessed are presented in the synoptic Table 1, Table 2 and Table 3 respectively. The electronic databases including Pub Med, Scopus, Google, and Google scholar were searched with the keywords “hepatoprotective”, “hepatotoxicity”, “liver toxicity” or “liver injury” with “plant”, “extract”, “herb”, or “phytochemical”, “natural product”, “small molecules” with “acetaminophen”, and “paracetamol”. Table 4 in this review enlists all of the plants extract that showed hepatoprotective in experimental models of APAP-induced liver toxicity. The name of the medicinal plants has been arranged following the data retrieved from PubMed/Medline, Google Scholar, Science direct, and Scopus. We did not elaborate the detailed investigations performed with all these plant extracts herein as we focus mainly on phytochemicals which could be stepping stone for the drug discovery. Nearly all of the plant extracts have been reported hepatoprotective based on the biochemical and histopathological assessments of liver injury and protection. Briefly, these natural drugs appear hepatoprotective by restoring the antioxidant defense, preventing the occurrence of oxidative stress and subsequently curbing mitochondrial dysfunction and inflammation, as well as limiting the resultant necrotic cell death [25,26]. The phytochemicals that are enumerated in this review have shown to attenuate liver injury either in the in vitro; cell-based assays (microsomes or cell lines) or in vivo; in rats and mice models of liver toxicity in preclinical studies. In next paragraphs, each phytochemical has been discussed emphasizing their source, chemical name and their effect as well as underlying mechanism in countering APAP-induced liver toxicity. 

### 1.1. Acanthoic Acid 

Acanthoic acid, which is a pimarane-type diterpene, chemically known as [(1R,4aR,7S)-7-ethenyl-1,4a,7-trimethyl-3,4,6,8,8a,9,10,10a-octahydro-2H-phenanthrene-1-carboxylic-acid] or (−)-primara-9(11),15-dien-19-oic acid is obtained from the bark of *Acanthopanax koreanum* Nakai roots and *Croton oblongifolius* stems. Wu et al. (2010) have shown that pretreatment with acanthoic acid restored liver enzymes, improved antioxidants and inhibited lipid peroxidation in addition to histological salvage [27]. Further, it inhibited apoptosis, as shown by amelioration of hypoxia inducible factor-1α (HIF-1α) and caspase-3 in liver tissues [28]. 

### 1.2. Ajoene

Ajoene, an allylsulfur compound, chemically known as [(E)-1-(prop-2-enyldisulfanyl)-3-prop-2-enylsulfinylprop-1-ene)], is isolated from processed garlic in an *E*/*Z*-mixture [29]. Ajoene dose dependently inhibited depletion of thiol content and GSH from liver and restored the liver enzymes in mice model of APAP-induced hepatotoxicity [30]. 

### 1.3. Alpha Hederin

α-Hederin, an oleanane-type saponin is present in many plants including *Nigella sativa* and *Hedera helix*, is reputed for its benefits in respiratory diseases. α-Hederin was found to attenuate hepatotoxicity in mice induced by several liver toxicants including APAP, by dose dependently suppressing CYP450, CYPB5, CYP1A, CYP2A and CYP3A enzymes as well as NADPH-cytchrome-*C*-reductase activity in liver microsomes. α-Hederin also reduced activities of 7-ethoxyresorufin-*O*-dealkylation, 7-pentoxyresorufin-*O*-dealkylation, coumarin-7-hydroxylation, 7-ethoxycoumarin-*O*-deethylation, caffeine-N3-demethylation, chlorzoxazone-6-hydroxylation and the oxidation of testosterone to 2α-,6α-,15α-,15β-,16α-,16β-, and 18/12 α-hydroxyltestosterone, androstenedione, and 6-dehydroxytestosterone [31].

### 1.4. Amyrin

Amyrin, a triterpene exists as anisomeric mixture of α and β-amyrin in the resin exudate of *Protium heptaphyllum*. Amyrin isomers showed to ameliorate liver toxicity by the restoration of liver enzymes and GSH levels in the liver, along with histopathological salvage and reduced mortality. The effects were found to be comparable to NAC [32].

### 1.5. Andrographolide

Andrographolide, which is a diterpene lactone chemically known as (3E,4S)-3-[2-[(1R,4aS,5R,6R,8aS)-6-hydroxy-5-(hydroxymethyl)-5,8a-dimethyl-2-methylidene-3,4,4a,6,7,8-hexahydro-1H-naphthalen-1-yl]ethylidene]-4-hydroxyoxolan-2-one is isolated from *Andrographis paniculata*, a reputed natural remedy in traditional Chinese and Indian medicine. Handa and Sharma (1990) have reported its hepatoprotective activity against APAP and galactosamine-induced hepatotoxicity for the first time in rats [33]. Visen et al. (1993) further showed the protective effects in rat hepatocytes and found to be more efficacious than silymarin [34]. Roy et al. (2013) had developed nanoparticles of andrographolide incorporated in PLGA along with heparin. These nanoparticles found to be more bioavailable inliver tissues and protected mouse liver against APAP by rapid restoration of antioxidants and GSH content [35].

### 1.6. Anthocyanins

Anthocyanins belong to a group of natural pigments that were isolated from the dried calyx of *Hibiscus sabdariffa* L. was found protective against APAP-induced hepatotoxicity in rats by improving liver enzymes but failed to improve the liver histology [36]. The anthocyanin isolated from purple-fleshed sweet potato was also reported to attenuate hepatotoxicity in mice. It restored liver enzymes, improved antioxidant enzymes, inhibited lipid peroxidation and diminished the depletion of GSH from liver. The improved histology and dose-dependent reduction of CYP2E1, as well as CYP2E1-dependent aniline hydroxylation further showed the hepatoprotective effect. It also showed free radical scavenging activity and antioxidant action against ferric chloride and ascorbate-induced lipid peroxidation in mouse liver [37]. In another study, anthocyanin fraction exhibited antioxidant and free radical scavenging activity and improved histology, along with favourable modulation of numerous signaling pathways in hepatoprotection [38].

### 1.7. Apigenin

Apigenin, which is a flavone chemically known as 4′,5,7-trihydroxyflavone is abundantly found in numerous edible plants, such as parsley, oranges, grapefruit, celery, onions, thyme, lemon balm, chamomile, and wheat sprouts. It is reputed for its organoprotective properties and also found hepatoprotective in mice model of APAP-induced toxicity by salvaging liver tissues, restoring antioxidants, liver enzymes and GSH content along with the inhibition of lipid peroxidation [39].

### 1.8. Arjunolic Acid

Arjunolic acid, which is a triterpene chemically known as 2,3,23-Trihydroxyolean-12-en-28-oic acid is obtained from the bark of *Terminalia arjuna* and is present in the plants of the Combretaceae family. Arjunolic acid reported hepatoprotective in chemical induced hepatotoxicity and hepatocarcinogenesis models [40]. Ghosh et al. (2010) have reported that it prevented GSH depletion from liver and APAP metabolite formation by inhibiting the specific forms of CYP450 that aid in the metabolic activation of APAP to *N*-acetyl-*p*-benzoquinone-imine [41]. Further, it prevented the dissipation of mitochondrial membrane potential, release of cytochrome-C along with decreased activation of JNK and mitochondrial permeabilization as well as downstream Bcl-2 and Bcl-xL phosphorylation.

### 1.9. Berberine

Berberine, which is an alkaloid chemically known as 5,6-dihydro-9,10-dimethoxybenzo(g)-1,3-benzodioxolo(5,6-a) quinolizinium sulfate is obtained from several dietary plants, including *Berberis aristata* and it is widely studied for its pharmacological properties, including hepatoprotective. For the first time, Janbaz and Gilani (2009) showed its hepatoprotective effects against APAP-induced hepatotoxicity [42]. It also increased pentobarbital-induced sleeping time and strychnine-induced toxicity that indicated its inhibitory effect on microsomal drug metabolizing enzymes, CYPs [42]. Recently, Vivoli et al. (2016) have demonstrated its effect in various experimental models like APAP-induced liver toxicity, methionine, and choline deficient diet induced steatohepatitis and in cultured murine macrophages [43]. Berberine also reduced mortality, restored liver enzymes, and inhibited the inflammasomes components, the major mediator of inflammation which requires activation of the cytokines IL-1β and IL-18 that are generated upon caspase-1 activation. The activation of inflammasomes in APAP-induced hepatotoxicity seems to be a major mediator in hepatocyte injury, immune cell activation and amplification of inflammation and cell death. Thus, inhibiting activation of P2X_7_, the purinergic receptors that mediates inflammasome activation by berberine appears a novel approach [43].

### 1.10. Bixin

Bixin, a group of carotenoids extracted from the seeds of *Bixa orellana* (Annatto) is a FDA approved popular food additive and colorant in cosmetics. It showed potent antioxidant and anti-inflammatory properties and protection against DNA damage and lipid peroxidation in liver. Rao et al. (2014) showed that solid lipid nanoparticle formulation of bixin exhibit sustained release following the first order diffusion kinetics and non-Fickian type of release mechanism. The in vivo studies revealed that the hepatoprotective property followed by the localization of bixin nanoparticals in liver tissues of rats [44].

### 1.11. Boswellic Acid

Boswellic acid, which is a mixture of triterpenic acids is chemically known as [(3R,4R,4aR,6aR,6bS,8aR,11R,12S,12aR,14aR,14bR)-3-hydroxy-4,6a,6b,8a,11,12,14b-heptamethyl-2,3,4a,5,6,7,8,9,10,11,12,12a,14,14a-tetradecahydro-1H-picene-4-carboxylic acid)]. It is principal constituent in the oleo gum resin of *Boswellia* species such as *B carteri, B serrata* and *B sacra*. Boswellic acid was found bioavailable in liver tissues following the oral ingestion. It protected against APAP-induced hepatotoxicity in mice by improving glutathione redox, inhibiting oxidative stress and attenuating pro-inflammatory cytokines and chemokines along with histopathologic salvage. It produced restoration of glutathione reductase (GR) and heme oxygenase-1 (HO-1) activities and inhibited CYP2E1 concomitant with reduced expression of toll-like receptors; TLR-3 and -4, MyD88, NF-κBp50, NF-κB p65 and JNK in liver tissues [45]. 

### 1.12. Brusatol

Brusatol, which is a natural quassinoid terpenoid, chemically known as methyl 13,20-epoxy-3,11,12-trihydroxy-15-((3-methyl-1-oxo-2-butenyl) oxy)-2,16-dioxopicras has been isolated from the fruit of *Brucea javanica*. Recently, Olayanju et al. (2015) found that brusatol treatment attenuated nuclear factor-like 2 (Nrf2) signaling following post-transcriptional mechanism in mouse hepatoma Hepa-1c1c7 cells [46]. Italso sensitized these cells to the chemical insult induced by the hepatotoxic metabolites of APAP including 2,4-dinitrochlorobenzene, iodoacetamide and *N*-acetyl-*p*-benzoquinone imine. The inhibitory effects were found to be independent of its repressor kelch-like ECH-associated protein-1 (Keap1), the proteasomal and autophagic protein degradation system and protein kinase signaling pathways that reveal the novel roles of Nrf2 regulation.

### 1.13. Caffeic Acid

Caffeic acid, which is a polyphenolic compound chemically known as trans-3,4-dihydroxycinnamic acid is abundantly found in edible plants including many fruits, coffee, and honey. For the first time, caffeic acid was showed to attenuate liver toxicity by restoring liver enzymes in rats [47]. In another study, caffeic acid showed to attenuate APAP-induced liver injury by restoring GSH and liver enzymes as well as reducing myeloperoxidase (MPO) activity, ROS levels and histopathologic salvage. It was also improved cell viability and suppressed ROS formation in L-02 cells from normal human liver and HepG2 cells. Further, it enhanced expression of endogenous antioxidants such as Nrf2, HO-1 and NAD(P)H:quinone oxidoreductase 1 (NQO1) and reduced expression of Keap1 there by prevented the binding of Keap1 to Nrf2 and thus activating Nrf2 in hepatocytes. The in silico data showed the interaction of Nrf2 binding site in the Keap1 protein and the in vitro study showed minimal effect on the enzymatic activity of CYP3A4 and CYP2E1 [48]. In another in vitro and in vivo study, authors showed the hepatoprotective mechanism of caffeic acid by down-regulating mRNA expression and transcriptional activation of early growth response-1. Caffeic acid also reduced the expression of growth arrest and DNA-damage-inducible protein (Gadd45) α and inhibited activation of extracellular-regulated protein kinase (ERK1/2) signaling cascade. Altogether, the studies reveal the inhibitory effect of caffeic acid on ERK1/2-mediated Egr1 transcriptional activation that is attributed to the detoxification of APAP-induced liver injury [49]. 

### 1.14. Calamusins 

Calamusins compounds are isolated from the ethanol extract of rhizomes of *Acorus calamus*. Calmusins A to H are the sesquiterpenes, while calamusin-I is a norsesquiterpene. Calamusin C, D, F and I (10 μM) have been reported to exert hepatoprotective activity against APAP-induced toxicity in HepG2 cells [50].

### 1.15. Carnosic Acid

Carnosic acid, which is a phenolic diterpene chemically known as (4aR,10aS)-5,6-dihydroxy-1,1-dimethyl-7-propan-2-yl-2,3,4,9,10,10a-hexahydrophenanthrene-4a-carboxylic acid) is isolated from the leaves of *Rrosmarinus officinalis* (Rosemary) and Sage. Guo et al. (2016) have shown that carnosic acid exert hepatoprotective effects by restoring liver enzymes and reducing liver necrosis [51]. Further, it was found to inhibit lipid peroxidation, pro-inflammatory cytokines and chemokines and phosphorylated IκBα and p65 proteins in the liver and suppressed cleaved caspase-3, Bax and phosphorylated JNK protein expression. It also facilitated Nrf2 translocation into nucleus through blocking interaction between Nrf2 and Keap1 that resulted in the up-regulation of antioxidant genes. However, in another study, Dickmann et al. (2012) showed that carnosic acid did not exhibit significant time dependent inhibition for any of the cytochrome P450 enzymes in primary human hepatocytes and human liver microsomes [52]. It dose dependently induced CYP2B6 and CYP3A4 and inhibited CYP2C9 and CYP3A4 enzymes catalyzed reactions. The increase in the activities of CYP2B6 and CYP3A enzymes were found to be comparable to phenobarbital and rifampicin, respectively. Though, the safety needs to be confirmed further. 

### 1.16. Chlorogenic Acid

Chlorogenic acid, the esters of caffeic and quinic acid are abundantly found in numerous plants and consumed through diet or beverages. Zheng et al. (2015) showed its hepatoprotective effect by the inhibition of pro-inflammatory cytokines, MPO expression and activity, restoration of liver enzymes and salvage of liver tissues along with diminution of raised expression of TLR-3, TLR-4 and MyD88 and the increased phosphorylation of inhibitor of kappa B (IκB) and p65 subunit of NF-κB in liver [53]. Further, it also improved cell viability in L-02 cells and showed the inhibition of activities of CYP2E1 and CYP1A2 in addition to improved antioxidant signals against APAP-induced cytotoxicity [54]. 

### 1.17. Chrysin

Chrysin, which is a flavone chemically known as 5,7-dihydroxy-2-phenylchromen-4-one is abundantly found in many plants, including fruits, vegetables and mushrooms. Chrysin was found an inhibitor of sulfo-conjugation of APAP by human liver cytosol with IC_50_ values < 1 µM. The inhibitory actions were attributed to the presence of 7-hydroxyl group in structure [55]. In another study, Morimitsu et al. (2004) have shown that chrysin elicit inhibitory effects on sulfo- and glucurono conjugation of APAP in rat cultured hepatocytes and liver subcellular preparations [56]. Recently, chrysin was reported as modulator of intestinal *P*-glycoprotein (*P*-gp) and drug-metabolizing enzymes that plays an important role in the first-pass-metabolism and pharmacokinetics of APAP in the in vitro non-everted gut sacs preparation and rats [57]. It was observed that chrysin increases the systemic exposure of APAP which needs to be studied in detail for clear conclusive remarks. 

### 1.18. Corynoline, Acetylcorynoline and Protopine 

Corynoline, acetylcorynoline or protopine were found to ameliorate liver injury in mice and liver microsomes with a pronounced efficacy of acetylcorynoline than corynoline and protopine. These compounds also showed biphasic response (inhibition followed by induction) on P450 in mice liver [58]. In another study, protopine was found to attenuate APAP-induced hepatotoxicity and suppress microsomal enzymes in rats. Protopine restored the liver enzymes mediating the inhibition of microsomal drug metabolizing enzymes [59].

### 1.19. Curcumin

Curcumin, a yellow polyphenol pigment chemically known as (1E,6E)-1,7-bis(4-hydroxy-3-methoxyphenyl) hepta-1,6-diene-3,5-dione is the main bioactive constituent in the rhizomes of *Curcuma longa*, popularly known as turmeric and reputed for its use in dietary, culinary, and cosmetic purposes. Several in vitro and in vivo studies have demonstrated that the protective effects of curcumin against liver injury mediating attenuation of oxidative stress, inflammation, and cell death. The cytoprotective effect against APAP was demonstrated in rat hepatocytes by attenuating lipid peroxidation, but no effect was found on depletion of lactae dehydrogenase (LDH) and GSH and in time dependent action at low concentrations. However, higher doses have shown the protective effects [60]. The effects were confirmed in vivo in rats, wherein it dose-dependently attenuated liver and renal toxicity by improving antioxidants, restoring liver enzymes and salvage histology. It potentiated the protective effects of NAC and also reduced the therapeutic dose of NAC [61,62]. In another study, curcumin provided protection against genomic instability, cell death, and oxidative stress in the liver. It was found to restore liver enzymes, inhibit lipid peroxidation, modulate APAP-induced alterations in genes expression of antioxidant and inflammatory cytokines, matrix metalloproteinase, DNA fragmentation and apoptosis [63]. The hepatoprotective effects were further reconfirmed in mice models [64,65,66].

### 1.20. Diallyl Sulfide

Diallyl sulfide, which is chemically known as 3-prop-2-enylsulfanylprop-1-ene is isolated from garlic known to impart flavor to garlic. It has been demonstrated to protect against APAP-induced liver toxicity as evidenced by restoration of liver enzymes and reduction in mortality in a time- and dose-dependent manner. In liver microsomes, it showed the hepatoprotective effect by the inhibition of APAP metabolism [67]. In another study, diallyl sulfone, a metabolite of diallyl sulfide was shown to protect against APAP-induced liver toxicity in mice by improving histopathology, liver enzymes and restoration of hepatic GSH levels. It also suppressed oxidative APAP metabolites in the plasma with no effect on non-oxidative metabolites of APAP. It repressed the rate of APAP oxidation to *N*-acetyl-*p*-benzoquinone imine, a glutathione conjugate by inhibiting CYP2E1 activity in liver microsomes [18]. Further, the organosulfur compounds of garlic were reported to protect against hepatotoxicity by inhibiting P450-mediated APAP bioactivation. The presence of *S*-allyl pharmacophore seems to confer the CYP2E1 inhibitory property to the sulfide compounds of garlic [68]. Diallyl sulfide gets converted into diallyl sulfoxide and diallyl sulfone by CYP2E1 and all of these are competitive inhibitors of CYP2E1. They also believed to induce other CYPs and phase II enzymes as well as augments enzymatic and non-enzymatic hepatic antioxidants [69]. 

### 1.21. Dioscin

Dioscin, a steroid saponin chemically known as 3-*O*-[α-l-Rha-(1->4)-[α-l-Rha-(1->2)]-β-d-Glc]-diosgenin is abundantly found in dietary plants *Dioscorea pseudojaponica*. Zhao et al. (2012) reported that dioscin in HepG2 cells attenuates mitochondrial impairment and cell death and improves cell viability [70]. The in vivo study showed similar effects and proteomic analysis revealed that *Suox*, *Krt18*, *Rgn*, *Prdx1*, *MDH* and *PNP* proteins were involved in the hepatoprotection. Additionally, it decreased expression of ATP2A2 and mitochondrial cardiolipin and regulated Ca^2+^ levels in mitochondria by attenuating and CYP2E1 activation. Further, it also modulated apoptotic proteins Bcl-2, Bid, Bax, Bak and p53 and activated aryl hydrocarbon receptor (AhR).

### 1.22. Diosmin 

Diosmin, a flavanoid chemically known as 3’,5,7-Trihydroxy-4’-methoxyflavone 7-rutinoside is abundantly found in citrus fruits and available clinically for the management of venous insufficiency. Diosmin was found to attenuate APAP-induced liver toxicity by inhibiting GSH depletion from liver and improving the enzymes activating glutathion-*s*-transferases permitting the captation of the reactive metabolites of the APAP and other liver toxicants [71].

### 1.23. (−)-Epigallocatechin-3-gallate 

(−)-Epigallocatechin-3-gallate, a polyphenolic compound is one of the most abundant catechin in tea with numerous pharmacological properties and therapeutic benefits. EGCG was found to curb metabolism and toxicity of APAP in rats by restoring liver enzymes, suppressing the activities of hepatic CYP3A, CYP2E1, uridine diphosphate glucuronosyltransferase and sulfotransferase. It also reduced APAP-glucuronate and -glutathione contents in plasma and liver [72].

### 1.24. Esculetin

Esculetin, which is a coumarin class of polyphenolic compound is chemically known as 6,7-dihydroxycoumarin and abundantly found in many medicinal plants, including *Artemisia capillaries*, *Artemisia scoparia*, *Citrus limonia*, *Ceratostigma willmottianum*, *Cichorium intybus* and *Bougainvllra spectabillis*. Gilani et al. (1998) have reported that esculetin restored the liver enzymes and reduce mortality in mice [73]. The inhibition of lipoxygenase pathway is believed to account for hepatoprotective action of esculetin. 

### 1.25. Ferulic Acid

Ferulic acid, which is a polyphenolic compound with structural resemblance to curcumin is abundantly found in leaves and seeds of many vegetables, fruits and cereals such as brown rice, whole wheat, and oats. Wand and Penf (1994) for the first time demonstrated the hepatoprotective activity of sodium ferulate, an active ingredient of *Angelica sinensis* Diels [74]. Sodium ferulate restored the liver enzymes and improved glutathione redox cycles and antioxidants, along with inhibition of lipid peroxidation in mice. Recently, ferulic acid dose-dependently restored liver enzymes, improved antioxidants, inhibited pro-inflammatory cytokines, *TLR4* expression and p38 mitogen-activated (MAPK), and activation of *NF-κB* in mice. The hepatoprotective effects involve inhibition of TLR4-mediated inflammatory responses and the expression of CYP2E1 [75].

### 1.26. Fulvotomentosides

Fulvotomentosides are the total saponins that were obtained from the flower extracts of *Lonicera fulvotomentosa*. Fulvotomentosides were found to ameliorate APAP-induced hepatotoxicity in mice by restoration of liver enzymes and reduced hepatic CYP450, CYPB5, and NADPH-cytochrome-C reductase. They exhibited reduced APAP-glutathione level, increased hepatic glucuronyltransferase activity and increased urinary elimination of APAP-glucuronide, with no effect on liver UDP-glucuronic acid in mice microsomes. This indicated that detoxification involves CYP450 and glucuronidation of APAP [76]. In another study, fulvomentosides showed similar hepatoprotective effects against hepatotoxicity induced by APAP [77]. Another derivative of fulvotomentoside known as sapindoside B ameliorated APAP-induced hepatotoxicity in mice by salvaging liver tissues and preventing GSH depletion and restoring liver enzymes along with reduction in mortality. It also promoted urinary excretion of APAP that is attributed to the suppression of hepatic CYP450 [78]. 

### 1.27. Galangin

Galangin, a flavonoid that is chemically known as 3,5,7-trihydroxyflavone is widely found in many plants including *Alpinia officinarum* and *Helichrysum aureonitens*. Galangin showed hepatoprotective effect in mice against propacetamol, a water soluble derivative of APAP. Galangin attenuated oxidative stress, increased GSH levels and inhibited microsomal CYP2E1 levels in the liver and found more potent than silymarin and NAC. However, galangin did not reduce mortality significantly [79].

### 1.28. Gallic Acid

Gallic acidand its derivatives are polyphenolic compounds that were widely distributed in different parts of plants and fruits, thus being often consumed directly or indirectly by humans as food stuffs and preservatives etc. Rasool et al. (2010) reported its hepatoprotective effects against hepatotoxicity in mice by restoring liver enzymes, inhibiting lipid peroxidation and pro-inflammatory cytokines and enhancing antioxidant defense by improving glutathione redox cycle [80]. 

### 1.29. Genistein

Genistein, a phytoestrogen and isoflavone that is chemically known as 4’,5,7-trihydroxyisoflavone is abundantly found in numerous edible plants including soybeans and mainly varieties of pulses. Genistein was found bioavailable in liver and amelioratedlipid peroxidation and restored liver enzymes by modulating APAP biotransformation. It also accelerated and promoted APAP glucuronidation by activating UGTs and glutathione peroxidase and inhibiting CYP2E1 [81]. Genistein get metabolized by CYP1A2 and CYP2E1 and CYP1A2 was predominantly responsible for 3’-OH-genistein formation; primary metabolite of genistein since its formation was inhibited [82]. Genistein was showed to reduce the formation of sulphate derivative of APAP and its raised excretion into bile arises from the inhibition of sinusoidal efflux transport [83]. Recently, genistein showed to inhibit APAP-induced cytotoxicity in fetal hepatocyte cell line (L-02), HepG2 and Hep3b cells, as evidenced by improved antioxidants, cell viability, hepatic enzymes and GSH redox in a dose-dependent manner. It also enhanced the metabolic transformation of APAP to glucuronic acid inL-02, HepG2, and Hep3b cells via the Nrf2/Keap1 pathway [84].

### 1.30. Geranylgeranylacetone 

Geranylgeranylacetone, which is an acyclic polyisoprenoid chemically known as 6,10,14,18-tetramethyl-5,9,13,17-nonadecatetraen-2-one is reputed as an anti-ulcer agent with minimal adverse effects. It has been shown to ameliorate liver necrosis by inhibiting lipid peroxidation and myeloperoxidase activity as well as restoring liver enzymes. However, it did not suppress hepatic CYP2E1 activity nor prevent depletion of GSH contents from liver [85].

### 1.31. Gingerol

6-Gingerol, which is chemically known as (5S)-5-hydroxy-1-(4-hydroxy-3-methoxyphenyl) decan-3-one is one of the major bioactive component of a widely used plant; *Zingiber officinalis*. It was reported to restore the liver enzymes, correct total bilirubin, inhibits lipid peroxidation and normalizes antioxidant status in liver in mice model of hepatotoxicity and found to be comparable to the standard drug, silymarin [86].

### 1.32. Ginkgolide

Ginkgolide A, a terpenic lactone that is chemically known as 9H-1,7a-(Epoxymethano)-1H,6aH-cyclopenta(c)furo(2,3-b) is abundantly present in the leaves of *Ginkgo biloba* that is widely-used herbal dietary supplement for its effects on health promoting and therapeutic benefits. The extract contains bilobalide and Ginkgolide A which played different roles in the modulation of CYP2B1 and CYP3A23 gene expression and enzyme activities. Ginkgolide A showed its protective effects on APAP toxicity in hepatocytes isolated from adult male Long-Evans rats. Ginkgolide A was found to increase CYP3A23 mRNA levels and CYP3A mediated enzyme activity that in part account to the potentiating effect on APAP toxicity. Whereas, other derivatives, such as ginkgolide B, ginkgolide C, ginkgolide J, quercetin, kaempferol, isorhamnetin and isorhamnetin-3-*O*-rutinoside failed to affect LDH leakage that is caused by APAP [87].

### 1.33. Glycyrrhetinic Acid

Glycyrrhetinic acid isomers are pentacyclic triterpenoid isolated from the roots of licorice plant; *Glycyrrhiza glabra* and possess various pharmacological properties such as antioxidant, antitumor and anti-inflammatory activities. Liu et al. (1994) for the first time reported that glycyrrhizin, 18 α-glycyrrhetinic acid and 18 β-glycyrrhetinic acid treatment were found to protect mice against APAP and other liver toxicants. In addition to histopathological salvage, these compounds restored serum activities of liver enzymes and sorbitol dehydrogenase [31]. Glycyrrhizin was also found to affect glucuronidation in the liver by increasing the activities of *p*-nitrophenol UDP-glucuronosyltransferase (UGT), known as UGT1A, which is indicative of its detoxificating property of xenobiotics [88]. Lin et al. (1997) have reported that hepatoprotective effect of *Scutellaria rivularis* Benth fractions known as Ban-zhi-lian against APAP and other toxicant models of liver toxicity while using glycyrrhizin as standard reference medicine [89]. In an in vivo study using metabolomics, glycyrrhetinic acid showed to protect against APAP by histological salvage and restoration of liver enzymes [90]. 

### 1.34. Glycyrrhizin

Glycyrrhizin or glycyrrhizic acid, a pentacyclic triterpenoid glycoside, chemically known as 29-Hydroxy-11,29-dioxoolean-12-en-3-yl 2-*O*-hexopyranuronosylhexopyranosiduronic is one of the bioactive constituent in roots of *Glycyrrhiza glabra*, popularly known as licorice. It is one of the highly consumed herbs and is widely studied for its therapeutic benefits in experimental and human studies. For the first time, Liu et al. (1994) in a preliminary study reported its hepatoprotective activity against APAP [31]. It has been suggested to detoxify xenobiotics by activating glucuronidation via increasing UGT and intracellular concentrations of hepatic UDP-glucuronic acid in rat liver [88]. In another study, Wan et al. (2009) reported that glycyrrhizin in combination with matrin that is extracted from *Sophora flavescens* [91]. It reduced mortality in APAP-induced hepatotoxicity in mice through immunosuppressive properties and inhibiting inflammation that was further supported by improved liver function and histology.

### 1.35. Gomisin A

Gomisin A, which is a lignan compound chemically known as 5,6,7,8-tetrahydro-1,2,3,12-tetramethoxy-6,7-dimethyl-10,11-methylenedioxy-6-dibenzo(a,c)cyclooctenol is isolated from *Shizandra* fruits. GomisinA was found to restore the liver enzymes, inhibit lipid peroxidation and reduce the necrotic changes in liver, as examined in histological and biochemical analysis [92]. In addition, similar results were observed in another study wherein it reported to suppress lipid peroxidation and induce hepatocyte growth factor [93]. 

### 1.36. Guajavadimer A 

Guajavadimer A, a dimeric monoterpenoid of sesquiterpene origin consisting of two caryophyllenes, a benzylphlorogulcinol and a flavonone-fused structure is isolated from the leaves of *Psidium guajava* L. Guajavadimer A in a preliminary study in HepG2 cells showed to attenuate APAP-induced liver toxicity [94]. 

### 1.37. Hesperidin

Hesperidin, a biflavonoid and flavanone glycoside consisting of the flavone hesperitin bound to the disaccharide rutinose, which is chemically known as 3’,5’-ihydroxy-4’-methoxy-7-rutinosyloxyflavan-4-on, is found in highly nutritious foods such as oranges, tangelos, tangerines, grapefruits, and other citrus fruits. Hesperidin showed hepatoprotective property in many experimental models, including APAP-induced hepatotoxicity [95]. It has been found to restore the levels of antioxidant enzymes and serum levels of liver enzymes and it prevents apoptotic death and inflammatory cytokines. 

### 1.38. Homopterocarpin

Homopterocarpin, which is an isoflavonoid chemically known as (6aS,11aS)-3,9-dimethoxy-6a,11a-dihydro-6H-[1]benzofuro[3,2-c]chromene is obtained from the ethanolic extract of stem bark of *Pterocarpus erinaceus* Poir. It was found to restore liver enzymes, inhibit lipid peroxidation and restore antioxidants in liver and corrected altered liver function [96].

### 1.39. Hyperoside

Hyperoside, which is a flavonol glycoside chemically known as (2-(3,4-dihydroxyphenyl)-5,7-dihydroxy-3-[(2S,3R,4S,5R,6R)-3,4,5-trihydroxy-6-(hydroxymethyl)oxan-2-yl]oxychromen-4-one) is obtained from *Hypericum perforatum*, *Crataegus oxycantha*, and *Apocynum venetum* L. Xie et al. (2016) showed that hyperoside dose dependently ameliorated lipid peroxidation, oxidative and nitrosative stress and increased activities and expression of uridine diphoshate glucuronosyltransferases and sulfotransferases [97]. It was also found to inhibit CYP2E1 activities that attrbute to the APAP detoxification.

### 1.40. Isoquercitrin

Isoquercitin or hirsutrin, a naturally occurring glycoside of quercetin is chemically known as (2-(3,4-dihydroxyphenyl)-5,7-dihydroxy-3-[(2S,3R,4S,5S,6R)-3,4,5-trihydroxy-6-(hydroxymethyl) oxan-2-yl]oxychromen-4-one). Xie et al. (2016) demonstrated the hepatoprotective effect of isoquercitrin, as evidenced by the amelioration of oxidative/nitrosative stress and inflammation by blocking the NF-κB and MAPK pathways [98]. It was also found to restore the liver enzymes and diminish centrilobular necrosis by regulating the activities of sulfotransferases and CYP2E1 that enhances hepatic detoxification of APAP. In a recent study, a microbiota-derived metabolite of quercetin; 3,4-dihydroxyphenylacetic acid has also been found to restore liver enzymes, attenuate lipid peroxidation, augment antioxidants and salvage the histology. It was found to promote Nrf2 translocation to the nucleus and enhance the expression of phase II enzymes and antioxidant enzymes that promotes APAP detoxification [99].

### 1.41. Isorhamnetin

Isorhamnetin, a polyphenolic metabolite of quercetin that is chemically known as quercetin-3-methyl-ether is isolated from the leaves of *Cistus laurifolius Linn.* In a preliminary study, it has been reported to restore liver enzymes, improve GSH content in liver and inhibit lipid peroxidation in plasma and liver in mice model of hepatotoxicity [100].

### 1.42. Kaempferol Derivatives

Kaempferol8-*C*-β-galactoside, a congener of kaempferol is isolated from extract of *Solanum elaeagnifolium*. It was shown to protect against APAP-hepatotoxicity by improving liver enzymes and salvaging liver tissues, comparable to silymarin [101]. Another kaempferol derivative known as kaempferol-3,7-dimethyl-ether is isolated from the extracts of leaves of *Cistus laurifolius* L. It was also found to improve cellular GSH levels, inhibit lipid peroxidation in plasma and liver and restore liver enzymes in mice model [100].

### 1.43. Lophirones

Lophirones are chalcone dimers that are isolated from stem bark of *Lophira alata* and reported to exhibit antioxidant, chemopreventive, antimutagenic, anticarcinogenic and hepatoprotective activity. Recently, Ajiboye (2016) demonstrated the hepatoprotective effect of lophirone B and C in mice by restoration of liver enzymes, enzymatic and non-enzymatic antioxidants along with attenuation of oxidative stress, pro-inflammatory cytokines, lipid peroxidation and reduced formation of conjugated dienes, protein carbonyl, lipid hydroperoxides, and fragmented DNA [102].

### 1.44. Lupeol

Lupeol, a pentacyclic triterpenoid that is chemically known as (1R,3aR,5aR,5bR,7aR,9S,11aR,11bR,13aR,13bR)-3a,5a,5b,8,8,11a-hexamethyl-1-prop-1-en-2-yl-1,2,3,4,5,6,7,7a,9,10,11,11b,12,13,13a,13b-hexadecahydrocyclopenta[a]chrysen-9-ol) is abundantly found in several dietary plants such as Crataeva, Mango, andOlive etc. Kumari and Kakkar (2012) have demonstrated its hepatoprotective activity against APAP-induced hepatotoxicity in rat hepatocytes [102]. It inhibited lipid peroxidation, ROS generation, and mitochondrial depolarization and restored liver enzymes as well as antioxidants and has shown the improved viability of hepatocytes. It also inhibited DNA damage and cell death by preventing downregulation of Bcl-2, upregulation of Bax, release of cytochrome-C, and the activation of caspase 9/3. The protective effects were further confirmed in vivo based on the attenuation of oxidative stress and histological salvage [103].

### 1.45. Luteolin

Luteolin, a flavone that is chemically known as 2-(3,4-dihydroxyphenyl)-5,7-dihydroxychromen-4-one is predominantly found in many plants, fruits and flowers and are reputed for its health benefits including liver diseases. Luteolin was found to inhibit sulfation in isolated liver cytosolic and microsomal preparations [56]. Recently, Tai et al. (2015) showed its antioxidant and anti-inflammatory activities against APAP in mice [104]. Luteolin restored liver enzymes, augmented the endogenous antioxidant defense, and inhibited lipid peroxidation and endoplasmic reticulum stress and GSH depletion from liver. It also inhibited pro-inflammatory cytokines and inflammatory mediators including iNOS, TNF-α, NF-κB, and nitrotyrosine. The inhibition of conjugation depends on both C5 and 7 hydroxyl substitutions on the A-ring of the flavone structure. Another derivative, luteolin 7-*O*-β-galacturonyl-(2-->1)-*O*-β-galacturonide, a new digalacturonide flavone is isolated from extract of flowers of *Lantana camara*, elicited potent hepatoprotective activity against APAP. It exerted free radical scavenging and antioxidant activity and restored the liver enzymes, along with histological salvage of liver tissues [105]. Luteolin-7-glucoside that was also isolated from the plant, *Glossogyne tenuifolia* Cassini was found to elicit hepatoprotection against APAP in BALB/c mice mediating antioxidant activity [106].

### 1.46. Magnolol

Magnolol, a biphenolic compound which is chemically known as (2-(2-hydroxy-5-prop-2-enylphenyl)-4-prop-2-enylphenol), is isolated from the bark of *Magnolia officinalis* is widely used in traditional Chinese and Japanese medicines. It has been shown to inhibit CYP1A and 2C in rats with no effect on CYP3A and play a role in the metabolic balance of lipids through liver X receptor α. Chen et al. (2009) have demonstrated the hepatoprotective activity of magnolol on APAP-induced hepatotoxicity in the rats by improving antioxidants, liver enzymes and ameliorating of lipid peroxidation along with liver tissues salvage [107].

### 1.47. Meso-Zeaxanthin

Meso-zeaxanthin, a xanthophyll carotenoid, is not a constituent of a normal human diet but comprises one-third of the primate macular pigment rarely found in diet and is believed to be formed at the macula by metabolic transformations of ingested carotenoids. Meso-zeaxanthin along with lutein and zeaxanthin known as macular pigment is believed to protect against age-related macular degeneration and is reputed nutrient for eye health. Lutein and zeaxanthin are obtained from dietary sources such as green leafy vegetables and orange and yellow fruits and vegetables. It was found to augment antioxidants, normalize GSH levels and restore liver enzymes along with histologic salvage against APAP and other liver toxicants in rats [108].

### 1.48. Methoxypsoralen

5-Methoxypsoralen, a naturally occurring linear furocoumarin chemically known as 4-Methoxy-7H-furo[3,2-g]chromen-7-one is obtained from the essential oils of bergamot, and citrus fruits, including grapefruits. It has been reputed in therapeutics for its use in combination with ultraviolet A irradiation to manage psoriasis and vitiligo. It was found to ameliorate liver necrosis by reducing the infiltration of inflammatory cells and dose dependent inhibition of lipid peroxidation, restoration of liver enzymes, and normalization of glutathione ratio [109].

### 1.49. Methyl Sulfonylmethane

Methyl sulfonylmethane, a sulfur rich compound that is commonly present in many dietary plants consumed as grains, fruits, vegetables and beverages. Bohlooli et al. (2013) have reported that it prevented APAP-induced liver toxicity in rats due to its antioxidant and sulfur donating properties. It also prevented lipid peroxidation, MPO formation and GSH depletion from liver and restored liver enzymes along with improving antioxidants [110].

### 1.50. Morin

Morin, a flavonoid that is chemically known as (2-(2,4-dihydroxyphenyl)-3,5,7-trihydroxychromen-4-one), is isolated from many fruits including *Maclura pomifera* (Osage orange), *Maclura tinctoria* (old fustic), and from the leaves of *Psidium guajava* (guava). Morin treatment ameliorated liver necrosis by reducing release of HMGB1, NALP3 and caspase-1 along with histological salvage and restoration of liver enzymes [111]. It also strengthened cellular defense by the attenuation of oxidative stress induced deactivation of Akt (Ser473) causes suppression in GSK3β and Fyn kinase activation. It regulated PHLPP2 activity by suppressing Nrf2 ubiquitination and enhanced nuclear Nrf2 retention as well as ARE-Nrf2 binding affinity.

### 1.51. Naphthoflavone

β-naphthaflavone, a synthetic derivative of a naturally occurring flavonoid is a ligand of the aryl hydrocarbon receptor, which mediates the potent activation of CYP1A. It caused a potentiation of APAP toxicity and/or death of both obese and lean Zucker rats. APAP overdose produced reduction of hepatic cytochrome P450 enzyme-substrate activities in lean Zucker rats. However, obese Zucker rats are less affected by the hepatotoxic effects of APAP overdoses [112].

### 1.52. Naringenin

Naringenin, a flavonoid aglycone of naringin that is chemically known as 5,7-dihydroxy-2-(4-hydroxyphenyl) chroman-4-one is commonly found in citrus fruits. Recently, it was found hepatoprotective against APAP in metallothionein null mice. Naringenin inhibited lipid peroxidation, normalized gluathione redox and restored liver enzymes along with improved histopathology [113]. It did not inhibit DNA and protein synthesis [114]. However, it has been reported to cause a weak inhibition of APAP oxidation [115].

### 1.53. Oleanolic Acid

Oleanolic acid (OA), a pentacyclic triterpene chemically known as 3-β-3-Hydroxyolean-12-en-28-oic acidis abundantly found in medicinal plants used in traditional Chinese medicine. It has been found to exhibit potent anticancer, anti-osteoporosis, antiobesity, antidiabetic, antihyperlipidemic, anti-inflammatory, antioxidant, and immunoregulatory and hepatoprotective effects. Liu et al. (1993) for the first time demonstrated its hepatoprotective property against APAP in mice as evidenced by improved antioxidant defense and restoration of liver enzymes in liver [116]. It did not affect liver UDP-glucuronic acid concentration, but it increased hepatic glucuronosyl transferase activity toward APAP. Further, it was found to increase hepatic metallothionein levels in Cd/hemoglobin assay and appears protective against hepatotoxicants, such as d-galactosamine plus endotoxin, thioacetamide, furosemide, colchicine, carbon tetrachloride, APAP, cadmium and bromobenzene [31]. In another study, OA was showed to ameliorate hepatotoxicity induced by chemical toxicants, including APAP [77]. The authors showed that OA decreased mouse liver CYP1A and CYP2A enzymes with minimal effect on CYP3A enzymes. It also increased GSH content in liver without affecting GSH peroxidase and GSH reductases [117]. The hepatoprotective activities were further confirmed as OA prevented APAP-induced the overproduction of NO and decline in GSH levels in liver along with reduced mortality [118]. Mechanistically, OA enhanced the expression of metallothionein, *Nrf2*, NQO1, HO-1, and glutamate-cysteine ligases (Gclc and Gclm) in liver and induced genes that were involved in proliferation with the suppression of P450 genes against hepatotoxicants [119]. Reisman et al. (2009) reconfirmed that OA protect liver by Nrf2-dependent and Nrf2-independent mechanism following the nuclear accumulation of Nrf2 that leads to the induction of Nrf2-dependent genes and contributes in hepatoprotection [120]. Recently, 2-cyano-3,12 dioxooleana-1,9-diene-28-imidazolide (CDDO-Im) a more efficacious and potent triterpenoid derivative from OA was synthesized [121]. It has been shown as a potent antioxidant and anti-inflammatory agent by diminishing iNOS production and activating the Nrf2-Keap1 pathway.

### 1.54. Paenol

Paenol, chemically known as 2’-hydroxy-4’-methoxyacetophenone is isolated from the root bark of *Paeonia spp.* and popular as *Moutan cortex* root in traditional Chinese medicine. Paeonol treatment attenuated lipid peroxidation, liver necrosis, and restored the liver enzymes, as well as antioxidants in liver along with inhibition of APAP-induced phosphorylated JNK protein expression without affecting p38 and Erk1/2 [122]. Moreover, it also prevented against APAP-induced cytotoxicity in primary mouse hepatocytes evidenced by the attenuation of pro-inflammatory cytokines and ROS formation along with suppression of IKKα/β, IκBα, and p65 phosphorylation. All of these mechanisms were attributed to the hepatoprotective effect of paenol.

### 1.55. Panaxatriol

Panaxatriols are the saponin constituents mainly isolated from *Panax notoginseng* which is major source of ginsenosides consists of two groups based on the types of the panaxadiol group (e.g., ginsenoside-Rb1 and -Rc) and the panaxatriol group (e.g., ginsenoside-Rg1 and -Re). The ginsenosides are widely studied for their therapeutic potential mediating multiple pharmacological properties. It has been shown that the ginsenoside-Rg1 and -Re had no CYP3A inhibitory effect [123], thus it may be devoid of ginseng-drug interaction. Wang et al. (2014) reported that panaxatriol inhibited pro-inflammatory cytokines, restored the thioredoxin-1 expression, an important redox regulator that play an important role in countering oxidative stress and subsequent inflammation [124]. Panaxatriol also inhibited apoptosis by regulating pro-caspase-12 expression. Also, ginsenoside-Rg3 enhanced the GSH content and multidrug resistance-associated protein expression in NAPQI induced rat hepatocytes [125].

### 1.56. Procyanidins

Procyanidins are the polymeric flavan-3-ols isolated from skin of *Prunus amygdalus* popularly known as almond, a dietary nut that has shown to exhibit antioxidant, anti-inflammatory, antiatherosclerotic and anticancer properties. It has been shown to enhance the expression of *Nrf2* and antioxidant respons element (ARE) reporter gene activity in HepG2 cells and induce the expression of phase II enzymes includingNQO1, catalase, glutathione peroxidase, and superoxide dismutase. In APAP-induced hepatotoxicity in mice, it attenuated hepatotoxicity through the activation of Nrf2/ARE-mediated phase II detoxifying/antioxidant enzymes [126].

### 1.57. Pterostilbene

Pterostilbene, a dimethylated resveratrol derivative that is chemically known as 4’-Hydroxy-3,5-dimethoxy-trans-stilbene, is mainly found in blueberries and are found to show potent pharmacological actions, including antioxidant, anti-inflammatory, and anti-apoptotic and therapeutic benefits in liver diseases. El-Sayed et al. (2015) have shown that pterostilbene exerted hepatoprotective effect against APAP-induced hepatotoxicity by restoring liver enzymes, inhibiting pro-inflammatory cytokines and lipid peroxidation and augmenting antioxidant activity along with the suppression of cell death [127]. Further, the hepatoprotective effects were affirmed by histopathological preservation and they were found to be comparable to silymarin.

### 1.58. Punicalagin and Punicalin

Punicalagin, an ellagitannin polyphenolic compound is abundantly found in fruit, husk and juice of pomegranate. Punicalagin and punicalin were also extracted from the leaves of a Combretaceous plant, *Terminalia catappa*. They exhibited multiple pharmacological properties such as neuroprotective, cardioprotective and hepatoprotective due to potent antioxidant and anti-inflammatory properties. Lin et al. (2001) have shown that hepatoprotective property of punicalagin and punicalin were exhibited through restoration of the liver enzymes and the inhibition of lipid peroxidation, along with improved antioxidant defense against APAP-induced hepatotoxicity in rats [128]. The histopathological salvage further confirmed the protective effects, though at high doses they appear hepatotoxic.

### 1.59. Quercetin

Quercetin is one of the most popular polyphenolic flavonol type compound reported to contain many phenol structural units. It predominantly found in glycosides form in large number of dietary plants including fruits, vegetables, beverages, spices and ornamental plants. Till date, it is extensively studied for its health and therapeutic benefits in experimental and clinical studies and it is considered as one of the highly consumed dietary flavonoid in day to day life across the world. Gilani et al. (1997) first reported the hepatoprotective activity of quercetin [129]. It was shown to reduce APAP-induced liver toxicity by promoting the repletion of GSH and the enzymes activating glutathione-*s*-transferases permitting the captation of the reactive metabolites of the APAP and other liver toxicants [71,130]. Quercetin was found to attenuate liver toxicity by restoring liver enzymes in rats [49]. Another derivative, quercetin-3,7-dimethyl-ether which was isolated from leaves of *Cistus laurifolius* L. has been shown to protect APAP-induced liver toxicity by antioxidant action [102]. Quercetin was found to ameliorate APAP-induced liver injury by restoring liver enzymes and antioxidants, inhibiting lipid peroxidation concomitant to histological salvage and correcting alter liver function tests similar to the standard drug, NAC [62]. It was also shown to ameliorate hepatorenal toxicity in rats by attenuating oxidative and nitrosative stress in liver and kidney and improving mitochondrial energy production [131]. However, recently, quercetinand chrysin have been shown to enhance the systemic exposure of APAP by inhibiting intestinal *P*-glycoprotein and metabolism of APAP [57]. In an approach to improve the drug delivery of quercetin, quercetin loaded self-nanoemulsifying drug delivery system was developed, which protected the liver injury. The optimized quercetin formulation was shown to enhance solubility and dissolution and it displayed potent protection by biochemical and histopathological improvement against APAP-induced hepatotoxicity in the form of free radical scavenging, antioxidant augmenting, and antilipiperoxidative activity [132]. In another recent study, quercetin inhibited APAP-induced cytotoxicity in human liver cells mediating *Nrf2* antioxidative signaling pathway inducing *p62* expression, inhibiting the binding of *Keap1* to *Nrf2* in L-02 cells. It enhanced the nuclear translocation of Nrf2 and induced the expression of the ARE-dependent genes like catalytic or modify subunit of glutamate-cysteine ligase (Gclc/Gclm), and HO-1. Docking studies indicated that the interaction of quercetin with the Nrf2-binding site in Keap1 protein, but it did not affect Keap1 expression. It also enhanced the expression of p62 and p62 siRNA and activated JNK in hepatocytes [133].

### 1.60. Resveratrol

Resveratrol, a polyphenol compound of stilbene group that is chemically known as 3,4,5-trihydroxystilbene, is abundantly present in grapes, berries, nuts and beverages. It is one of the comprehensively studied compounds for health benefits and pharmaceutical development. A convincing number of experimental studies [134,135,136,137,138] along with some detailed reviews [139,140] in the past few years reported the benefits of resveratrol in liver diseases. Sener et al. (2006) for the first time demonstrated its hepatoprotective property against APAP-induced liver toxicity in mice [138]. Resveratrol was found to attenuate hepatotoxicity by inhibiting the activation of pro-inflammatory cytokines, oxidative stress, lipid peroxidation and myeloperoxidase activity. The restoration of liver enzymes and histological preservation of liver tissues further confirmed the hepatoprotective effects of resveratrol due to its potent antioxidant and anti-inflammatory properties [138]. In another study, resveratrol treatment was found to be protective against APAP-induced liver injury in CD-1 mice with an observation that Th1-dominant response in Th1/Th2 cytokine balance and TNF-play an important role in APAP-induced liver injury [137]. Du et al. (2015) investigated the hepatoprotective mechanism and showed that resveratrol did not affect the formation of reactive metabolites, protein bindings and JNK pathway. It was found to inhibit downstream nuclear DNA fragmentation and release of apoptosis-inducing factor and endonuclease G from mitochondria independent of Bax pore formation along with reduction in protein nitration following APAP challenge due to scavenging of peroxynitrite [136]. In another study, resveratrol was found to inhibit bioactivation of APAP by suppressing activation of CYP2E1, CYP3A11, and CYP1A2 activities and inducing Sirtuin 1 activation; an important player in energy metabolism and regulates cell cycle, apoptosis, and inflammation. Further, sirtuin activation negatively regulated p53 signaling to induce cell proliferation-associated proteins including cyclin D1, cyclin dependent kinase 4, and proliferating cell nuclear antigenand facilitated hepatocyte proliferation. It also inhibited the activation of JNK pathway and protected against mitochondrial injury [135]. The sirtuins mediated hepatoprotective effects were further confirmed in vivo and in vitro models of APAP-induced hepatotoxicity. Resveratrol was found to increase APAP-reduced SIRT1 activity comparable to the selective synthetic sirtuins activators [134]. Taken together, the studies are suggestive of hepatoprotective properties of resveratrol in APAP-induced liver toxicity and the activation of sirtuins appear to be a novel mechanism of hepatoprotection.

### 1.61. Rhein

Rhein, an anthraquinone glycoside, which is chemically known as 4,5-dihydroxy-9,10-dioxoanthracene-2-carboxylic acid, is abundantly found in many plants including *Rheum palmatum* L., *Aloe barbadensis* Miller, *Cassia angustifolia* Vahl, and *Polygonum multiflorum* Thunb. Rhein has been shown potent antioxidant, anti-inflammatory, antitumor, neuroprotective, and hepatoprotective properties. It has been reported to confer protection dose dependently against APAP-induced liver and renal toxicity in rats, by normalizing antioxidants, restoring liver enzymes and GSH levels along with suppressed lipid peroxidation and histological salvage due to potent antioxidant action [141].

### 1.62. Rutin

Rutin or vitamin P or quercetin-3-*O*-rutinoside is a polyphenolic bioflavonoid that is chemically known as 3,3’,4’,5,7-pentahydroxyflavone-3-rhamnoglucoside and abundantly found in vegetables, beverages, and dietary plants, including *Artemisia scoparia*. Its cytoprotective effect, such as gastroprotective, hepatoprotective, and anti-diabetic has been shown via antioxidant, anti-inflammatory, and organoprotection in several studies. It has been showed to reduce mortality and restore liver enzymes [142].

### 1.63. Saikosaponin D

Saikosaponin D, which is chemically known as β-d-Galactopyranoside, (3β,4α,16α)-13,28-epoxy-16,23-dihydroxyolean-11-en is a major constituent isolated from *Bupleurum falcatum* that is popularly used for liver diseases in eastern Asian countries. Liu et al. (2014) reported that Saikosaponin D protected against APAP-induced hepatotoxicity by down-regulating *NF-κB* and STAT3-mediated inflammatory signaling as evidenced by decreased phosphorylation of *NF-κB* and signal transducer and STAT3 and suppressed *NF-κB* target genes such as pro-inflammatory cytokine *IL-6* and *Ccl2*, and *STAT3* genes such as suppressor of cytokine signaling 3 (*Socs3*) and fibrinogen gene analysis (*Fga, Fgb* and *Fgg*). Also, it increased the expression of anti-inflammatory cytokine IL-10 mRNA [143].

### 1.64. Salidroside

Salidroside or p-tyrosol, a phenylethanoid glycoside is chemically known as (2R,3S,4S,5R,6R)-2-(hydroxymethyl)-6-[2-(4-hydroxyphenyl) ethoxy]oxane-3,4,5-triol) is a major constituent of perennial flowering plant *Rhodiola* species mainly *Rhodiolarosea* and *Rhodiola imbricata, Rhodiola algida* and *Rhodiola crenulata*. In traditional medicine, it is used for the management of many chronic degenerative diseases and has shown numerous pharmacological properties including adaptogenic, neuroprotective, anti-tumor, cardioprotective, antidepressant, antioxidant, anti-inflammatory and hepatoprotective. Wu et al. (2008) have demonstrated that salidroside protects against APAP-induced hepatotoxicity by inhibiting lipid peroxidation, pro-inflammatory cytokines and restoring liver enzymes along with antioxidants [144]. It was also displayed histopathological salvage and suppression of caspase-3 and hypoxia inducible factor-1α (HIF-1α) expression in liver. The protective effects of salidroside were found to be comparable to that of NAC. In another study, Guo et al. (2014) developed and validated a simple and specific LC-MS/MS method for the determination of salidroside and its metabolite p-tyrosol in rat liver tissues that suggested its bioavailability in the liver tissues and its hepatoprotective effect [145].

### 1.65. Salvianolic Acids

Salvianolic acid B, a polyphenolic compound, is isolated from the aqueous factions of extracts of *Salvia miltiorrhiza* Bunge, popularly used in traditional Chinese medicine and represents one of the highly used medications with application from oral to intravenous. It is one of the most potent antioxidant, anti-inflammatory agent and reported to protect various organs, such as brain, heart, kidney, and liver from oxidative stress [146]. Salvianolic acid B was found to confer hepatoprotective effects against APAP by inducing Nrf2 expression [147]. Salvianolic acid B treatment restored liver enzymes, enhanced the expression of Nrf2, HO-1 and glutamate-l-cysteine ligase catalytic subunit (Gclc). Furthermore, it also activated the phosphatidylinositol-3-kinase (PI3K) and protein kinase C (PKC) signaling pathways. In another study, salvianolic acid B showed to maintain redox status and mitochondrial metabolic activity in rat hepatocytes, but fail to inhibit CYP2E1 [146]. Altogether, it appears that salvianolic B protects against liver toxicity via the activation of the PI3K and PKC pathways.

### 1.66. Saponarin

Saponarin, a favone glycoside that is chemically known as (5-hydroxy-2-(4-hydroxyphenyl)-6-[(2S,3R,4R,5S,6R)-3,4,5-trihydroxy-6-(hydroxymethyl)oxan-2-yl]-7-[(2S,3R,4R,5S,6R)-3,4,5-trihydroxy-6-(hydroxymethyl)oxan-2-yl] oxychromen-4-one) is naturally occurring apigenin-6-*C*-glucosyl-7-*O*-glucoside isolated from *Gypsophila trichotoma.* It has been reported to possess antihyperglycemic, antimicrobial, antioxidant, anti-inflammatory and hepatoprotective properties. Simeonova et al. (2013) reported hepatoprotective effects in the in vitro/in vivo studies. In isolated rat hepatocytes, saponarin dose dependently improved cell viability and antioxidant defense and inhibited lipid peroxidation as well as LDH leakage [148]. Similar results were replicated in vivo in addition to the histological salvage of liver tissues. However, no changes in phase I enzyme activities of Aniline 4-Hydroxylase (AH) and Ethylmorphine-N-Demethylase (EMND) and cytochrome P450 quantity were detected. The protective effects were comparable to silymarin.

### 1.67. Sauchinone

Sauchinone, a polyphenolic lignin isolated from *Saururus chinensis* exhibits potent antioxidant and anti-inflammatory activity and protects hepatocytes against iron-induced toxicity [149]. Kay et al. (2011), have shown that sauchinone attenuated APAP-induced liver injury and its protective mechanism as activating *Nrf2* through the *PKCδ-GSK3β* pathway [150]. In hepatocytes, sauchinone activated Nrf2, leads to increased nuclear accumulation of *Nrf2*, activation of *NQO1*- ARE reporter gene and glutamate-cysteine ligase and NQO1 protein that imparts the restoration of hepatic GSH content. Sauchinone also activated protein kinase C-δ (PKCδ) that enhanced Nrf2 phosphorylation with a reciprocal decrease in its interaction with Keap1 and activated Nrf2 phosphorylation. Further, it was also found to enhance the inhibitory phosphorylation of glycogen synthase kinase-3β (GSK3β), suppressing Nrf2 activity dependent on PKCδ activation.

### 1.68. Schisandrol Derivatives

Schisandrin A, schisandrin B, schisandrin C, schisandrol A, schisandrol B and schisantherin A are the lignan compounds isolated from *Schisandra sphenanthera*, a reputed herb in traditional Chinese medicine for the treatment of many diseases including liver. These derivatives have been shown hepatoprotective against APAP-induced liver toxicity in mice [151]. The protective effects of these compounds were evidenced by restoration of GSH in liver, inhibition of lipid peroxidation, and restoration of liver enzymes in a dose-dependent manner. They were also found to attenuate the enzymatic activities of CYP450 isoforms viz. CYP2E1, CYP1A2 and CYP3A11 and alter APAP bioactivation mechanism [151]. This results in the reduced formation of toxic intermediate N-acetyl-p-benzoquinone imine NAPQI-GSH in vivo and in vitro both. Among these derivatives, schisandrol B was studied extensively and showed to attenuate activation of p53 and p21 and promote liver regeneration along with enhancement in antiapoptotic proteins such as cyclin D1, PCNA and BCL-2. Further, in silico studies also demonstrated that schisandrol B interferes with CYP2E1 and CYP3A4 active sites [152]. Schisandrol B exhibited a significant protective effect toward APAP-induced liver toxicity, potentially through inhibition of CYP-mediated APAP bioactivation and regulation of the p53, p21, CCND1, PCNA, and BCL-2 to promote liver regeneration [153]. In a recent study, schisandrol B further showed to attenuate APAP-induced hepatotoxicity in mice by the activation of Nrf2/ARE pathway and the regulation of *Nrf2* target genes *Nrf2/ARE* signaling pathway [153]. Schisandrol B treatment ameliorated liver toxicity and increased the nuclear accumulation of Nrf2 as well as expression of Nrf2 downstream proteins, including Gclc, GSR, NQO1, GSTs, MRP2, MRP3 and MRP4 in APAP-treated mice. The mechanism was further confirmed in HepG2 cells. Based on these studies, all of the lignans appear promising with better potential of schisandrol B in reducing hepatotoxicity by improving antioxidant defense and inhibiting the CYP mediated bioactivation of APAP.

### 1.69. Sesamol

Sesamol, a flavonoid lignin that is chemically known as 1,3-benzodioxol-5-ol is obtained fromthe oil that was extracted from seeds of *Sesamum indicum*. Chandrasekaran et al. (2009) demonstrated the ameliorative effect of sesamol pretreatment against APAP by improved liver enzymes and the inhibition of free radicals generation and subsequent lipid peroxidation and centrilobular necrosis [154]. In another report, authors reconfirmed the findings and showed that hepatoprotective effects were comparable to NAC at the equimolar doses post-treatment [155].

### 1.70. Silybin

Silybin dihemisuccinate, which is a soluble form of the flavonoid silymarin, was found to prevent GSH depletion and inhibit lipid peroxidation in liver along with restoration of liver enzymes altered by APAP [156,157]. In another study, silybin inhibited lipid peroxidation in isolated rat hepatocytes [158]. Conti et al. (1992) demonstrated the hepatoprotective properties of silipide, a silybin-phosphatidylcholine complex abbreviated as IdB 1016. Silipide dose dependently ameliorated APAP-induced liver toxicity due to its antioxidant action and bolstering of RNA and resultant protein synthesis [159].

### 1.71. Sweroside

Sweroside, an iridoid glycoside that is chemically known as (3S,4R,4aS)-4-ethenyl-3-[(2S,3R,4S,5S,6R)-3,4,5-trihydroxy-6-(hydroxymethyl)oxan-2-yl]oxy-4,4a,5,6-tetrahydro-3H-pyrano[3,4-*c*]pyran-8-one, is isolated from the flower buds of *Lonicera japonica* Thunb and *Swertia pseudochinensis* Hara. It has been traditionally used in treatment of liver diseases and showed hepatoprotective in chemical models of liver injury. Its high bioavailability in liver tissues is attributed to its liver regenerating and hepatoprotective activity [160]. The metabolic profile revealed the presence of several phase I, phase II and aglycone-related metabolites in rat urine [161]. Though, in one preliminary study, Liu et al. (1994) did not find protective of sweroside against APAP [77].

### 1.72. Syringic Acid

Syringic acid, a naturally occurring phenolic compound that is chemically known as *O*-methylated trihydroxybenzoic acid, is abundantly found in many edible mushrooms and vegetable, food and beverages plants. Syringic acid possesses high proteasome inhibitory activity and showed to alleviate APAP-induced liver injury by improving enzymatic and non-enzymatic antioxidant defense and restoration of liver enzymes along with histopathological preservation of liver tissues [162].

### 1.73. Tannic Acid

Tannic acid, a polyphenolic compound is naturally occurring tannins and abundantly found in edible plants, including fruits, vegetables, tea, strawberries, beans, grapes, coffee, persimmons, cocoa, and nuts. Recently, tannic acid has been shown to be protective against APAP-induced hepatotoxicity [163]. It restored activities of antioxidant and liver enzymes and inhibited endothelin-1, nitric oxide and malondialdehyde formation. It also suppressed the activation of pro-inflammatory cytokines, and apoptotic mediators, such as *c-Fos*, *c-Jun*, *NF-κB (p65)* and caspase-3 and increased Bax along with decreased Bcl-2 and increased Nrf2 and HO-1. The histologic salvage of liver tissues reconfirmed the protective effects and anti-oxidant, anti-inflammatory, and anti-apoptotic effects were attributed to confer hepatoprotective effects [163].

### 1.74. Thymoquinone

Thymoquinone, a quinone compound that is chemically known as 2-methyl-5-propan-2-ylcyclohexa-2,5-diene-1,4-dione, is found abundantly in the oil from the seeds of *Nigella sativa* and represent one of the widely studied molecule. In a study, Nagi et al. (2010) first showed that thymoquinone dose-dependently protect against hepatotoxicity in mice by reversing rise in liver enzymes in serum, total nitrate/nitrite, lipid peroxide, and a fall in GSH and ATP in APAP-induced hepatotoxicity. Though, it did not affect the metabolic activation of APAP [164].

### 1.75. Withferin A

Withaferin A, a withanolide alkaloid that is chemically known as (4β,5β,6β,22*R*)-4,27-dihydroxy-5,6-22,26-diepoxyergosta-2,24-diene-1,26-di), is isolated from the leaves of *Withania somnifera* popularly known as ‘Indian ginseng’. Withaferin A showed protection against liver necrosis by decreasing the activities of liver marker enzymes and prevents lipid peroxidation by improving antioxidant statusin mice model of hepatotoxicity [165]. It also suppressed JNK activation, mitochondrial Bax translocation, nitrotyrosine production, and upregulated *Nrf2*, *Gclc* and *NQO1* expression as well as down-regulated pro-inflammatory cytokines. In AML12 hepatocytes, it also reduced H_2_O_2_-induced oxidative stress and necrosis.

### 1.76. Miscellaneous

The lignans, 2,4′-epoxy-8,5′-neolignans and 7,9′;7′,9-diepoxylignans isolated from extract of *Penthorum chinese* was shown its hepatoprotective activity in hepatocytes [166]. The isoflavonoid compounds, such as 7-hydroxy-4′,5,6-trimethoxyisoflavone, 7-hydroxy-5,6-dimethoxy-2′,3′-methylenedioxyisoflavone, and 5,6-dimethoxy-2′,3′-methylenedioxy-7-*C*-β-*d*-gluco-pyranosyl isoflavone isolated from the seeds of *Lepidium sativum* L., displayed the amelioration of APAP-induced hepatotoxicity in rats by augmenting the endogenous antioxidants, improving the liver enzymes along with salvage of liver tissues [167]. Myricetin was found to inhibit microsomal CYP2E1 and CYP3A activities, but others, such as tangeretin, quercetin, naringenin and nobiletin does not inhibit [115]. Thymol and carvacrol found to enhance antioxidant and free radical scavenging activity and reduce activation of pro-inflammatory cytokines that are comparable to NAC in HepG2 cells [168]. Oxymatrine does not confer protective effects against AP-induced hepatotoxicity [77]. In a preliminary study, several triterpenoids, such as oleanolic acid, ursolicacid, uvaol, alpha-hederin, hederagenin, glycyrrhizin, 18-α-glycyrrhetinic acid, 18-β-glycyrrhetinic acid, 19-α-hydroxylasiatic acid, 28-*O*-β-d-glucoside, and 19-α-hydroxyl Asiatic acid were evaluated against APAP-induced hepatotoxicity. Uvaol, hederagenin, 19 alpha-hydroxyl Asiatic acid, 28-*O*-β-d-glucoside and 19-α-hydroxyl asiatic acid had no effect on APAP-hepatotoxicity whereas, glycyrrhizin, 18-α-glycyrrhetinic acid, 18-β-glycyrrhetinic acid, alpha-hederin, ursolic acid and oleanolic acid has reduced APAP-induced hepatotoxicity [31]. Girish et al. (2009) reported the hepatoprotective activity of ellagic acid against APAP-induced acute hepatotoxicity in a preliminary study and found the effects were comparable to silymarin.The protective effects were mediated by antioxidant activity and the restoration of liver cytochrome P450 enzymes [169].

## 2. Discussions

As represented in the tables, a large number of phytochemicals (Table 1, Table 2 and Table 3), plant extracts (Table 4), and herbal formulations (Table 5) have been shown to ameliorate APAP-induced liver injury. The available experimental studies reveal that phytochemicals and plant extracts exert hepatoprotective effects against APAP-induced liver toxicity due to their multiple pharmacological properties, including anti-inflammatory, antioxidant and antiapoptotic. Among them, the majority of them are linked to cascades that are involved in oxidative stress, inflammatory cytokine signaling, and cell death [187,188]. Mechanistically, phytochemicals and plant extracts showed to restore antioxidant defense by preventing glutathione depletion, improving antioxidants enzymes along with attenuation of lipid peroxidation and subsequently limiting inflammation and cell death.

Many plant extracts have been shown to improve the endogenous enzymatic and non-enzymatic antioxidants to inhibit lipid peroxidation and the activation and release of pro-inflammatory cytokines concomitant with prevention of depletion of GSH from the liver. The cardinal characteristic of APAP-induced liver injury is massive retrograde degeneration of the liver tissues resulting in the loss of liver enzymes followed by depletion of GSH and lipid peroxidation and inflammation. The dramatic depletion of glutathione is known to be responsible for the clinical manifestation of hepatotoxicity. In majority of the studies, the hepatoprotective effects of plant extracts against APAP-induced liver injury were confirmed by liver function tests, as evidenced by the restoration of the liver enzymes and attenuation of the rise of liver enzymes in the serum concomitant improvement in cellular architecture and reduced liver necrosis. The whole plant extract known to have various phytoconstituents that act synergistically to enhance efficacy and prevent toxicity when it is used as an adjuvant along with the modern medicine [189]. Therefore, the synergy of phytoconstituents could be beneficial to enhance their efficacy. 

Several formulations containing plant extracts of a single plant or many plants known as polyherbal formulation are often available in the market for treating liver disorders [190,191,192,193,194,195,196]. The polyherbal or single herb or herbomineral formulations showed hepatoprotective effects are represented in Table 5. One such example for single herb formulation that is quite popular from traditional to modern medicine is silymarin, a reputed hepatoprotective herbal drug preparation containing a single herb, known as Milk thistle [197]. Whereas, Liv 52^®^ represents a popular polyherbal preparation for liver diseases. Though, the management of liver disorders by a simple and precise herbal drug is still an intriguing problem. Silymarin is an extract from the seeds of milk thistle mainly contains flavonolignan isomers such as silybin, isosilybin, silydianin and silychristin with silybin is the most potent constituent [198]. On oral administration, silymarin absorbs quickly and eliminates mainly through bile as sulphates and conjugates. Silymarin has been shown to protect numerous preclinical models of liver diseases due to its antioxidative, anti-lipid peroxidative, antifibrotic, anti-inflammatory, membrane stabilizing, immunomodulatory, and liver regenerating properties [199]. Clinically, silymarin is found to be useful in alcoholic liver disease, liver cirrhosis, Amanita mushroom poisoning, viral hepatitis, toxic and drug induced liver diseases and in also diabetic patients [200,201,202,203,204,205]. The safety and efficacy of herbal medicines either as monotherapy or as an adjunct to conventional therapy for hepatotoxicity appears to be favorable, as indicated by many studies [206,207,208,209], thus the plant extract are believed to hold a promise in the management of APAP-associated hepatotoxicity. However, it is important to note that, very few of these plant extracts have been showed to attenuate necrotic or apoptotic cell death cascades but favorably modulate the antioxidant signaling pathways that are mediated by *Nrf2* and *Keap1*, along with modulation of different kinases viz. *JNK* and *MAPK*. Further, very few of them demonstrated the prevention of the metabolic activation of APAP by suppressing CYP2E1, a plausible mechanism believed to alter the bioactivation of APAP. In studies employing phytochemicals/plant extracts to investigate APAP-induced hepatotoxicity, very few studies have been compared with silymarin as a positive reference phytochemical for the comparative evaluation of hepatoprotective effects [205,210,211,212]. This may be attributed to the huge variation observed in the dose (25–200 mg/kg) and the dosing regimen (1–15 days) of silymarin treatment.

The model systems adapted to evaluate hapatoprotective agents whether it is in vitro or in vivo have its own merits and demerits; however the choice of model system selection mainly depends on the goals of the particular experimental paradigm. Several in vitro assays invovling cell lines and in vivo animal models have been developed to understand the pathogenesis of APAP-induced liver toxicity and to investigate the hepatoprotective agents. Recently, Kuo et al. (2016) have suggested that the natural antioxidants tested in non-suitable animal models may prove efficacious but it will impose ambiguity when these preclinical data are used as a baseline to design clinical studies. Thus, careful selection of suitable animal model is imperative to carry out well-defined preclinical studies. One such example is using rat as a model animal to evaluate APAP-induced hapatotoxicity, which is relatively resistant to APAP toxicity [26]; therefore, any data generated using this model system will impose inherent experimental bias. Many in vitro studies using phytochemicals/plant extracts to investigate the hapatoprotective effects have employed human hepatoma cell lines (e.g., HepG2, Hep3B, Huh7). These cell lines lack CYP enzymes, which are involved in the formation of hepatotoxic metabolites of APAP [213] therefore, such studies lose the clinical relevance due to the inherent variation of cellular physiologic and biochemical system. The CYP enzyme isoforms, mainly CYP2E1 is responsible for the bioactivation of AAP in humans and animals, which may represent a therapeutic target for APAP-induced hepatotoxicity.

Among the in vivo models, APAP-induced hepatotoxicity in mice is considered to be one of the best physiologically and clinically relevant model systems that represent the most of the pathophysiological features of APAP toxicity in humans. Whereas, among the in vitro models screening agents in primary mouse hepatocytes is considered to be closest to in vivo settings [214,215]. Additionally, the HepaRG cells also mimic the pathological changes similar to humans with APAP toxicity (except the requirement for JNK) [216,217]. Freshly isolated primary human hepatocytes are considered as gold standard for drug toxicity studies, including APAP-induced hepatotoxicity [218]. Jeschke and colleagues comprehensively reviewed the pathogenesis of APAP-induced toxicity and suggested that it is vital to choose a right animal species/in vitro system, timing/doses of APAP, as well as the assessments of signaling events, metabolic activation and protein adduct formation, the role of lipid peroxidation, and the apoptotic/necrotic cell death to elucidate hepatoprotective mechanisms and provide correct conclusion by avoiding the potential bias and pitfalls in the evaluation of hepatoprotectants [12,219].

The available literature reviewed herein reveal that a large number of plants and phytochemicals mediating antioxidant and anti-inflammatory properties appear hepatoprotective in preclinical models of APAP-induced liver injury. About five hundred plant extracts and fifty phytochemicals have shown to be hepatoprotective in preclinical studies with negligible clinical data. A vast majority of them have been shown to be hepatoprotective based on the biochemical, morphological, and histopathological assessments. All of them were shown to restore the liver enzymes and also protect liver cellular architecture. These plants and phytochemicals may provide novel chemical entities for future drug discovery and development against APAP-induced liver toxicity. Among many phytochemicals showed hepatoprotective against APAP few of them found to inhibit CYP2E1 that could be promising for further evaluation in APAP-induced liver toxicity. Despite a large number of plant extracts being demonstrated as hepatoprotective, the use of medicinal plants may have many issues, such as lack of standardization, quality control, heavy metal contamination, and presence of bacterial toxins.

Though, a large number of plant extracts and phytochemicals have been demonstrated hepatoprotective against APAP-induced liver toxicity, but those that shown hepatoprotective in numerous model systems and their effect on APAP bioactivation by inhibiting CYP2E1 has been demonstrated that could be promising to investigate further in detail. Although, the present preclinical data are markedly speculative for clinical usage, but it could be substantial for further evaluation of these plants and phytochemicals in clinical settings provided their human safety.

## Figures and Tables

**Table 1:** Phytochemicals showed hepatoprotective effect in the mice model of acetaminophen-induced liver toxicity.

Phytochemical	Dose of Phytochemical	Dose of APAP and Route	Efficacy and Major Mechanisms	CYP2E1 Inhibition	References
Acanthoic acid	50, 100 mg/kg, *p. o*. 2h before APAP	300 mg/kg, *i. p.*	LFT, antioxidants, anti-inflammatory, antiapoptotic and antinecrotic	No	[27]
Ajoene	20,50,100 mg/kg, *p. o.*, 2 & 24 h before APAP	300 mg/kg, *p. o.*	LFT, GSH	No	[30]
Apigenin	100, 200 mg/kg	350 mg/kg, *i. p.*	LFT, antioxidants, H&E	No	[39]
Astaxanthin	30, 60 mg/kg, *p. o.* × 14 days	300 mg/kg, *i. p.*	LFT, antioxidants, pro-inflammatory cytokines, inhibition of JNK signal pathway and phosphorylation of ERK and P38	No	[170]
Baicalin	15, 30, 60 mg/kg, *p. o.*	300 mg/kg, *i. p.*	LFT, cytokines, H&E, decrease hepatic phosphorylated extracellular signal-regulated kinase expression	No	[171,172]
Berberine	1 or 5 mg/kg, *i. p*	500 mg/kg, *i. p.*	LFT, mortality, NLRP3 inflammasome pathway	No	[43]
Boswellic acid	0.05, 0.1% in diet × 4 weeks	400 mg/kg, *i. p.*	LFT, antioxidants, cytokines and chemokines, toll-like receptor signaling and H&E	Yes	[45]
Carnosic acid	100 mg/kg × 3 days	400 mg/kg, *i. p.*	LFT, antioxidants, Nrf2/Keap pathway, H&E	No	[51]
Chlorogenic acid	5, 10, 20 or 40 mg/kg × 7days	300 mg/kg, *i. g*	LFT, antioxidants, antiapoptotic, ERK1/2, JNK, p38 kinases mediated MAPK pathway	No	[173]
Chlorogenic acid	10, 20, 40 mg/kg at 1h after given AP	400 mg/kg, and another 3h later	LFT, MPO, H&E, pro-inflamatory cytokines, chemokines, TLR3/4 and NFκB signaling	No	[53]
Corynoline, acetylcorynoline and protopine	50, 100 mg/kg, 8 to 24 h before APAP	-	LFT, antioxidants	Yes	[58]
Esculentoside A	2.5 mg/kg, *i. p.* twice in a day	400, 900 mg/kg, *i. p.*	LFT, antioxidants, H&E, increases Nrf2 expression and phosphorylation of AMPK, Akt and GSK3β	No	[174]
Ferulic acid	30, 100 mg/kg, *p. o*., t.d. × 3 days	350 mg/kg, *i. p.*	LFT, antioxidants, H&E, MAPK and TLR4 pathway	Yes	[75]
Gallic acid	100 mg/kg, *i. p.* 30 min after APAP	900 mg/kg, *i. p.*	LFT, pro-inflammatory cytokines, antioxidants	No	[80]
6-Gingerol	30 mg/kg, 30 min after APAP	900 mg/kg	LFT, antioxidants, comparable to the standard drug silymarin	No	[86]
Glycyrrhetinic acid	500 mg/kg × 20 days before APAP	400 mg/kg, *i. p.*	LFT, metabolism pathway of fatty acids, palmtioylcarnitine and oleoylcarnitine	No	[90]
Glycyrrhizin	Oral, *i. p.* and *i. v.*	200-600 mg/kg, *i. p.*	LFT, antioxidants, pro-inflammatory cytokines, antiapptotic, H & E, only *i. p., i. v.* effective	Yes	[175]
Hyperoside	10, 50, 100 mg/kg, *p. o.* for 3 days before APAP	300 mg/kg, *i. p.*	LFT, antioxidants, Nrf2/Keap pathway, Phase II enzymes	Yes	[97]
Isoquercitrin	10, 20, or 50 mg/kg, *p. o*. for 3 days before APAP	300 mg/kg, *i. p.*	LFT, Pro-inflammatory cytokines, antioxidants, NF-κB/MAPK pathway	Yes	[98]
Kaempferoll8-C-β-galactoside and C-glycoside	25, 50, 75 mg/kg	500 mg/kg	LFT, H&E, comparable to silymarin	No	[101]
Luteolin and quercetin 3-β-d-glucoside	200, 400 mg/kg, *p. o.* for 14 days	2 g/kg, *p. o.* × 14 days	LFT, antioxidants, H&E	No	[176]
Lycopene	10, 100mg/kg, *p. o.*	500 mg/kg, *p. o.*	LFT, antioxidants, MMP-2, H&E, morphometry	No	[177,178]
Naringenin	200, 400, and 800 mg/kg, *p. o.*	250 mg/kg, *s. c.*	LFT, antioxidants, H&E	No	[113]
Paeonol	25, 50, 100 mg/kg, *p. o.*, 6 and 24 h before APAP	400 mg/kg, *i. p.*	LFT, antioxidants, chemokines and cytokines, JNK pathways	No	[122]
Fulvotomentosides, oleanolic acid, total saponins of *Panax japonicus* & *Panax* notoginseng, sweroside, oxymatrine, dimethyl dicarboxylate biphenyl,	-	-	LFT, H&E, Fulvomentosides found most potent, oleanic acid, total saponins of *Panax japonicus* and *Panax notoginseng* had moderate hepatoprotective effects, sweroside, oxymatrine and dimethyl dicarboxylate biphenyl had no effect on APAP toxicity	No	[77]
α-Hederin and sapindoside B	20 mg/kg, *s. c.* twice	-	LFT, H&E, mortality	No	[78]
Procyanidins	1 or 10 mg/kg, *p. o.*	300 mg/kg, *i. p.*	LFT, enhanced Nrf2/ARE activity and phase II detoxifying/antioxidant enzymes	Yes	[126]
Rutin	20 mg/kg, *p. o.*	640 mg/kg, *p. o.*	LFT, antioxidants	No	[142]
Sodium ferulate	100 mg/kg, *p. o.*, q.d. × 10 days	130 mg/kg, *i. p.*	LFT, antioxidants	No	[74]
Salidroside	50, 100 mg/kg 2 h before APAP	300 mg/kg, *i. p.*	LFT, pro-inflammatory cytokines, antioxidants, antiapoptotic, H&E, parallel with NAC	No	[144]
Salvianolic acid B	25 and 50 mg/kg, *i. g.* × 3 days	300 mg/kg, *i. g.*	LFT, antioxidants, Nrf2, HO-1 and Gclc activation of the PI3K and PKC pathways	Yes	[147]
Sauchinone	6 h after APAP	500 mg/kg, *i. p.*	LFT, antioxidants, H&E, Keap1/Nrf2 and GSK3β-PKCδ pathway	No	[150]
Schisandrol B	200 mg/kg, *p. o.* for 3 days before APAP	400 mg/kg, *i. p*.	LFT, H&E, antioxidants, Nrf2/ARE signaling pathway	No	[153]
Schisandrol B	6.25, 25 and 100 mg/kg for 7 days before APAP	400 mg/kg, *i. p.*	LFT, antioxidants, antiapoptotic (p53, p21, CCND1, PCNA, and BCL-2)	Yes	[152]
Schisandrin derivatives	200 mg/kg/day, *p. o.*	400 mg/kg, *i. p.*	LFT, antioxidants, H&E	Yes	[151]
Silipide	400 mg/kg, *p. o.*	-	LFT, antioxidant activities	No	[159]
Quercitrin	10, 50 mg/kg, *p. o.* × 7 days	300 mg/kg, *i. p.*	LFT, antioxidants and Nrf2/ARE, anti-inflammatory, MAPK pathways including ERK, JNK, and p38 MAPK, comparable to silymarin	No	[179]
Tannic acid	25, 50 mg/kg, *p. o.* × 3 days	400 mg/kg, *p. o.*	LFT, antioxidants, pro-inflammatory cytokines, H&E, suppressed c-Fos, c-Jun, NF-κB (p65) and caspase-3, regulated Bax/Bcl-2, Nrf2 and HO-1	No	[163]
Trans-anethole	62.5, 125, 250 mg/kg, *p. o.*	250 mg/kg, *p. o.* in mice	LFT, antioxidants, pro-inflammatory cytokines, morphometrics, H&E	No	[180]
Withaferin A	7 mg/kg, *p. o.* in Nrf2 KO mice	250 mg/kg, *i. p.*	LFT, Keap1-independent & Pten/PI_3_K/Akt-dependent	No	[181]

**Table 2:** Phytochemicals showed hepatoprotective effect in the rat model of acetaminophen-induced liver injury.

Phytochemical	Dose of Phytochemical	Route and Dose of APAP	Efficacy and Major Mechanisms	CYP2E1 Inhibition	References
Andrographolide	200 mg/kg, *i. p.*, 1, 4 & 7 h after APAP	3 g/kg, *p. o.*	LFT, H&E, antioxidants	No	[33]
Berberine	4 mg/kg; *p. o.* twice × 2 days or 4 mg/kg every 6 h	-	LFT, antioxidants	Yes	[42]
Chlorogenic acid	40 mg/kg *p. o.* × 7 days	300 mg/kg, intragastric	LFT, antioxidants LFT, antioxidants	Yes	[54]
Esculetin	6 mg/kg	640 mg/kg, *p. o.*	LFT, antioxidants	No	[73]
Gomisin A	50 mg/kg	750 mg/kg *i. p.*	LFT, antioxidants, antiapoptotic, H&E	No	[92]
Hesperidin	100, 200 mg/kg × 14 days	750 mg/kg, *p. o.*	LFT, antioxidants, antioapoptotic, H&E	No	[95]
Liquiritigenin & Schisandrin C derivative	*p. o.* or *i. v*., 2–4 days		LFT, H & E, liquiritigenin and combination showed protection while schisandrin C derivative failed	No	[182]
Lupeol	150 mg/kg, *p. o*. × 30 days	1 g/kg	LFT, antioxidants, antiapoptotic, H&E	No	[183]
Magnolol	0.01, 0.1, 1 µg/kg 0.5 h after APAP	500 mg/kg, *i. p.* × 8 and 24 h	LFT, H&E, antioxidants	No	[107]
Pterostilbene	50, 100 mg/kg, *p. o.* × 15 days before APAP	800 mg/kg, *i. p.*	LFT, lipid profiles, pro-inflammatory cytokines, antioxidants, antiapoptotitic, antifibrotic, comparable to silymarin	No	[127]
Punicalagin and Punicalin	1,5,12.5 or 25 mg/kg, *i. p.*	500 mg/kg, *i. p.*	LFT, antioxidants, H&E	No	[128]
Rutin	20 mg/kg, *p. o.* × 11 days	500 mg/kg *p. o.* from day 1–3 in rats	LFT, H&E, TEM, antioxidants, comparable to silymarin	No	[184]
Saponarin	80 mg/kg, *p. o.* × 7 days	600 mg/kg, *i. p.*	LFT, antioxidants, H&E	Yes	[148]
Silybin	-	-	LFT, GSH and lipid peroxidation	No	[157]
Syringic acid	25, 50 and 100 mg/kg *p. o.*	750 mg/kg *i. p.*	LFT, H&E, comparable to silymarin	No	[162]

**Table 3:** Phytochemicals showed hepatoprotective effect in the in vitro model of acetaminophen-induced liver injury.

Phytochemicals	Dose of Phytochemical	Cells and Dose of APAP	Efficacy and Major Mechanisms	CYP2E1 Inhibition	References
Andrographolide	0.75–12 mg/kg *p. o.* × 7 days	Rat hepatocytes	LFT, viability, more potent than silymarin	No	[34]
Lupeol	10 μM	Rat hepatocytes, APAP (675 μM)	Maintaining redox and preventing mitochondria-mediated apoptosis	No	[103]
Paeonol	20, 40, 80 μM	Mouse hepatocytes H_2_O_2_ or APAP	LDH, ROS and pro-inflammatory genes and reduced IKKα/β, IκBα and p65 phosphorylation	No	[122]
Silibin	25 μM	Rat hepatocytes, APAP (25–30 mM)	Inhibited APAP toxicity, prevented DNA strand breaks formation	No	[185]
CalamusinsA-I	10 μM	HepG2 cells	Weak hepatoprotective activities against APAP	No	[50]
α-Hederin	10, 30 µM/kg, *s. c.* × 3 days	Rat liver microsomes	Dose-dependent suppression of liver cytochrome P450 enzymes	Yes	[186]
Saponarin	60-0.006 μg/mL	Rat hepatocytes, APAP (100 μM)	Cell viability, LDH, GSH, MDA	Yes	[148]
Chlorogenic acid	1, 10, 25, 50 and 100 μM/L	L-02 cells	LFT, cell viability	Yes	[54]
Procyanidins	10, 25 and 50 μg/L	HepG2 cells	Enhanced phase II detoxifying and antioxidant enzymes and Nrf2/ARE activity	Yes	[126]
Thymol and carvacrol	25, 50 and 100 µM	HepG2 cells	Antioxidants, pro-inflammatory cytokines, comparable to NAC	No	[168]

**Table 4:** The medicinal plants showed to ameliorate the acetaminophen-induced hepatotoxicity in different models.

Plant Names	Plant Names	Plant Names	Plant Names	Plant Names
*Abelmoschus moschatus*	*Boswellia ovalifoliolata*	*Eugenia jambolana*	*Mucuna capitataRoxb.*	*Sargassum tenerrimum*
*Abutilon indicum*	*Boswellia serrata*	*Fagonia olivieri*	*Mucuna pruriens*	*Sargassum variegatum*
*Acacia auriculiformis*	*Brassica juncea Linn.*	*Fermented ginseng*	*Muntingia calabura*	*Schisandra chinensis*
*Acacia indica*	*Bridelia micrantha*	*Fermented red ginseng*	*Musa paradisiaca*	*Schoenoplectus grossus*
*Acathopanax senticosus*	*Bryophylum pinnatum*	*Ficus exasperate*	*Musanga cecropioides*	*Scutia myrtina*
*Achillea wilhelmsii C.*	*Bupleurus spp.*	*Ficus hispida Linn*	*Mussaenda erythrophylla*	*Senecio scandens*
*Acronychia laurifolia*	*Caesalpinia bonduc Linn.*	*Ficus microcarpa Linn.*	*Myrica rubra Sieb.*	*Sesamum indicum*
*Adansonia digitata Linn.*	*Caesalpinia gilliesii*	*Ficus mollis*	*Nasturtium officinale*	*Sida acuta Burm. f.*
*Adhatoda vasica*	*Cajanus cajan*	*Ficus religisoa Linn.*	*Nauclea latifolia*	*Silene aprica*
*Aegle marmelos*	*Cajanus indicus*	*Flos lonicerae*	*Nigella sativa*	*Silybum marianum*
*Agaricus blazei*	*Calotropis procera*	*Foeniculum vulgare*	*Ocimum gratissimum*	*Smilax zeylanica Linn.*
*Ageratum conyzoides*	*Camelia sinesis*	*Fumaria indica*	*Opuntia robusta*	*Solanum alatum*
*Alcea rosea*	*Capparis sepiaria L.*	*Fumaria officinalis*	*Opuntia streptacantha*	*Solanum fastigiatum*
*Alchornea cordifolia*	*Caralluma umbellate*	*Fumaria parviflora*	*Ornithogalum saundersiae*	*Solanum indicum*
*Allium cepa*	*Cardiospermum halicacabum*	*Ganoderma amboinense*	*Oroxylum indicum*	*Solanum nigrum*
*Allium sativum*	*Carica papaya*	*Garcinia indica*	*Osbeckia octandra*	*Sophora flavescens*
*Alnus japonica*	*Carissa carandas Linn.*	*Garcinia kola*	*Oxalis corniculata*	*Sphaeranthus indicus*
*Aloe barbadensis*	*Carum copticum*	*Genista quadriflora*	*Oxalis strictalinn*	*Swertia chirata*
*Aloe vera*	*Cassia fistula*	*Gentiana manshurica*	*Paederia foetida*	*Swertia longifolia Boiss*
*Alpinia galanga*	*Cassia occidentalis L*	*Glossogyne tenuifolia*	*Paeonia anomala*	*Swertia punicea*
*Alstonia scholaris R. Br.*	*Ceiba pentandra Linn.*	*Glycosmis arborea*	*Pandanus odoratissimus*	*Swietenia mahagoni L.*
*Amaranthus caudatus*	*Centaurium erythraea*	*Glycosmis pentaphylla*	*Parinari curatellifolia*	*Syzygium aromaticum*
*Ambrosia maritima*	*Chelidonium majus*	*Gongronema latifolium*	*Pavonia zeylanica*	*Taraxacum officinale*
*Amorphophallus paeoniifolius*	*Cichorium endivia*	*Gossypium herbacium*	*Penthorum chinese*	*Taraxacum syriacum*
*Andrographis paniculata*	*Cichorium glandulosum*	*Gymnaster koraiensis*	*Pergularia daemia*	*Telfairia occidentalis*
*Anisochilus carnosus*	*Cinnamomum tamala*	*Gymnosporia montana*	*Phyllanthus acidus*	*Tephrosia purprea*
*Annona muricata*	*Cinnamomum zeylanicum*	*Gynostemma pentaphyllum*	*Phyllanthus amarus*	*Terminalia chebula*
*Anoectochilus formosanus*	*Cistus laurifolius Linn.*	*Gypsophila trichotoma*	*Phyllanthus emblica*	*Terminalia paniculata*
*Apium graveolens Linn.*	*Citrullus colocynthis*	*Haplophylum tuberculatum*	*Phyllanthus maderaspatensis*	*Tetracera loureiri*
*Apocynum venetum Linn.*	*Citrus hystrix*	*Harungana madagascariensis*	*Phyllanthus niruri Linn.*	*Teucrium poliumgeyrii*
*Aquilegia vulgaris*	*Citrus maxima*	*Hedyotis corymbosa*	*Phyllanthus polyphyllus*	*Teucrium stocksianum*
*Arctium lappa Linn*	*Citrus microcarpa*	*Hemodiscus indicus*	*Phyllanthus urinariae*	*Thymus vulgaris*
*Argania spinosa*	*Clausena dentata*	*Hibiscus hispidissimus*	*Piper methysticum*	*Tinospora cordifolia*
*Artemisia absinthium*	*Cleome chelidonii*	*Hibiscus sabdariffa L*	*Piper puberulum*	*Tournefortia sarmentosa*
*Artemisia capillaris*	*Clerodendron Inerme*	*Hippocratea africana*	*Pisonia aculeate*	*Trianthema portulacastrum*
*Artemisia maritima*	*Clitoria ternatea Linn.*	*Hippophae rhamnoides*	*Pittosporum neilgherrense*	*Tribulus terrestris Linn.*
*Artemisia pallens Walls*	*Cnidoscolus aconitifolius*	*Holostemma ada Kodien*	*Plantago major*	*Trichopus zeylanicus*
*Artemisia sacrorumLedeb.*	*Coldenia procumbens*	*Hordeum vulgare Linn.*	*Platycodon grandiflorum*	*Trichosanthes dioica*
*Artemisia scoparia*	*Conyza bonariensis*	*Hypericum perforatum*	*Pleurotus ostreatus*	*Trichosanthes lobata*
*Artichoke*	*Copaiba oil*	*Indigofera tinctoria Linn.*	*Pluchea arguta*	*Tridax procumbens Linn*
*Asparagus falcatus*	*Cornus officinalisSieb.*	*Iris spuria*	*Plumbago zeylanica*	*Trifolium alexandrinum*
*Asparagus racemosus*	*Corylus avellana*	*Ixeris chinensis*	*Polyalthia longifolia*	*Ulva reticulata*
*Asteracantha longifolia*	*Costus igneus*	*Khaya gradifoliola*	*Polygonum odoratum*	*Urtica dioica*
*Astragalus corniculatus*	*Crataegus songarica*	*Khaya senegalensis*	*Pongamia pinnata*	*Uvaria afzelli*
*Astragalus persicus*	*Croton zehntneri*	*Kigelia africana*	*Porphyra yezoensis*	*Vernonia amygdalina*
*Astragalus tournefortii*	*Cucurbita pepo*	*Kohautia grandiflora*	*Pouteria campechiana*	*Vigna angularis*
*Atropa acuminata*	*Cuscuta australis*	*Kombucha tea*	*Premna tomentosa*	*Vitellaria paradoxa*
*Auricularia polytricha*	*Cuscuta chinensis*	*Lawsonia inermis*	*Prosopis africana*	*Vitex doniana*
*Averrhoa bilimbi*	*Cyathea gigantea*	*Leea asiatica*	*Prosopis farcta*	*Wedelia calendulacea*
*Averrhoa carambola*	*Cynanchum atratum*	*Leonotis nepetifolia*	*Psidium guajava*	*Wedelia paludosa*
*Azadirachta indica*	*Cynara scolymus*	*Lepidium sativum Linn.*	*Pterocarpus osun Craib*	*Woodfordia fruticosa*
*Azolla microphylla*	*Cyperus scariosus*	*Lopatherum gracile*	*Pueraria lobata*	*Ximenia americana Linn.*
*Baccharis dracunculifolia*	*Cyperus segetum*	*Lophira lanceolata*	*Pyropia yezoensis*	*Xylopia aethiopica*
*Baccharis trimera*	*Dalbergia paniculata*	*Lycopersicum esculentum*	*Raphanus sativus*	*Zea mays Linn.*
*Balanites aegyptiaca*	*Desmodium adscendens*	*Lycopodium clavatum*	*Rhazya stricta*	*Zingiber officinale*
*Barleria prionitis Linn.*	*Dicranopteris linearis*	*Malva sylvestris Linn.*	*Rhodiola imbricata*	*Zingiber zerumbet*
*Basella alba*	*Dioscorea alata Linn.*	*Mangifera india*	*Rosa damascena*	*Zizyphus jujube*
*Bauhinia purpurea*	*Ecballium elaterium*	*Markhamia platycalyx*	*Rosa laevigataMichx*	*Zizyphus spina*
*Berberis aristata*	*Echinophora platyloba*	*Maytenus emerginata*	*Rosmarinus officinalis*	
*Beta vulgaris*	*Eclipta alba Hassk.*	*Melastoma malabathricum*	*Rubia cordifolia*	
*Bidens pilosa Linn.*	*Embelia ribes*	*Mesona palustris BL*	*Salacia oblonga*	
*Bixa orellana Linn.*	*Enantia chlorantha*	*Momordica charantia*	*Salvia miltiorrhiza*	
*Blumea mollis*	*Entada africana*	*Monochoria vaginalis*	*Santallum album*	
*Boehmeria nivea*	*Epaltes divaricate*	*Moringa oleifera Lam.*	*Sargassum binderi*	
*Boerhaavia diffusa*	*Eucalyptus maculata*	*Moutan cortex*	*Sargassum polycystum*	

**Table 5:** The polyherbal or single herb formulations showed protective against APAP-induced liver toxicity.

S. No.	Polyherbal/Single HerbFormulation
1	999 Ganmaoling^®^
2	A formulation of *Andrographis paniculata, Tinospora cordifolia* and *Solanum nigrum*
3	A polyherbal formulation containing eight herbs; Vasaguduchyadi Kwatha^®^
4	A polyherbal formulation containing a mixture of leaves of *Gongronema latifolia*, *Ocimum gratissimum* and *Vernonia amygdalina*
5	A polyherbal formulation containing aqueous extracts of *Ocinnim larrilifolium*, *Crassocephaluin vitellitiurn*, *Guizotia scabra* and *Vernonia lasiopus*
6	A polyherbal formulation containing extracts of *Butea monosperma*, *Bauhinia variegata* and *Ocimum gratissimum*
7	A polyherbal formulation containing *Hydrocotyle asiatica*, *Tephrosia purpurea*, *Solanum nigrum*, *Citrullus colocynthis*, *Momordica charantia*
8	A polyherbal formulation HP-4^®^ is a combination of 80% alcoholic extract of leaves of *Aloe vera*, *Bacopa monniera*, *Moringa oleifera* and rhizome of *Zingiber officinale*
9	A polyherbal formulation, HD-03^®^
10	A polyherbal formulations containing five bioactive fractionated extracts of *Butea monosperma*, *Bauhinia variegata* and *Ocimum gratissimum*
11	A polyherbal formulation containing extracts of *Andrographis paniculata* Nees., *Phyllanthus niruri* Linn., *Phyllanthus emblica* Linn.
12	A polyherbal mixture of *Tinospora cordifolia*, *Boerhavia diffusa*, *Phyllanthus amaraus*, *Euphorbia hirta*, *Wedelia chinensis*
13	A polyherbal Siddha formulation, Karisalai Karpam^®^
14	A polyherbal Siddha medicine, Amukkara chooranam^®^
15	Ban-zhi-lian
16	Bazhen decoction
17	Biherbal formulations of *Aerva lanata* and *Achyranthes aspera*
18	Chai-Hu-Ching-Kan-Tang^®^
19	D-003^®^
20	DA-9601^®^, a quality-controlled extract of *Artemisia asiatica*
21	Fengxiang Yigankang^®^
22	Fourteen vitex honeys
23	Gn-3^®^, a stilbene polymer isolated from *Gnetum parvifolium*
24	Habb-e-Asgand^®^, polyherbal Unani formulation
25	Hepax^®^, a polyherbal formulation
26	Himoliv^®^, a polyherbal formulation
27	Huanglian-Jie-Du-Tang^®^
28	Hwang-hua-mih-tsay (*Wedelia chinensis*)
29	IH636 grape seed extract
30	Karisalai Karpam tablet^®^
31	Kava herbal dietary supplements
32	Liu weiwuling Tablets^®^
33	Livartho*^®^*, a polyherbal formulations consist of 10 active constituents of medicinal plants viz, *Andrographis paniculata, Cichorium intybus*, *Tephrosia purpurea*, *Solanum nigrum*, *Phyllanthus amarus*, *Tinospora cordifolia*, *Eclipta alba*, *Berberis aristata*, *Piper longum* and *Emblica officinalis*
34	Livina^®^, a polyherbal formulation
35	Majoon -e-Dabeed-ul-Ward
36	MAP, a Standardized Herbal Composition, Blend Comprising *Myristica fragrans*, *Astragalus membranaceus* and *Poriacocos*
37	Picroliv^®^
38	Polyherbal ayurvedic formulations, Liv 52^®^, Livergen^®^, Livokin^®^, Octogen^®^, Stimuliv^®^, Triphala^®^ and Tefroliv^®^, Tritone^®^ (Livosone)
39	Shekwasha^®^
40	Somanathitamrabhasma^®^, a tamra bhasma preparation containing shudhatamra, parada, gandhaka, haritala and manashila
41	‘Teng-khia-u’
42	Yang-Gan-Wan
